# Suppression of the human malic enzyme 2 modifies energy metabolism and inhibits cellular respiration

**DOI:** 10.1038/s42003-023-04930-y

**Published:** 2023-05-22

**Authors:** Ju-Yi Hsieh, Kun-Chi Chen, Chun-Hsiung Wang, Guang-Yaw Liu, Jie-An Ye, Yu-Tung Chou, Yi-Chun Lin, Cheng-Jhe Lyu, Rui-Ying Chang, Yi-Liang Liu, Yen-Hsien Li, Mau-Rong Lee, Meng-Chiao Ho, Hui-Chih Hung

**Affiliations:** 1 Department of Life Sciences, National Chung Hsing University, Taichung, 402 Taiwan ROC; 2 Ph.D. Program in Tissue Engineering and Regenerative Medicine, National Chung Hsing University, Taichung, 402 Taiwan ROC; 3 Institute of Biological Chemistry, Academia Sinica, Taipei, 115 Taiwan ROC; 4 Institute of Medicine, College of Medicine, Chung Shan Medical University, Taichung, 402 Taiwan ROC; 5 Instrument Center, Office of Research and Development, National Chung Hsing University, Taichung, 40227 Taiwan ROC; 6 Department of Chemistry, National Chung Hsing University, Taichung, 402 Taiwan ROC; 7 Institute of Biochemical Sciences, National Taiwan University, Taipei, 106 Taiwan ROC; 8 Institute of Genomics and Bioinformatics, National Chung Hsing University, Taichung, 402 Taiwan ROC; 9 Advanced Plant and Food Crop Biotechnology Center, National Chung Hsing University, Taichung, 402 Taiwan ROC

**Keywords:** Enzyme mechanisms, Biophysical chemistry, Structural biology

## Abstract

Human mitochondrial NAD(P)^+^-dependent malic enzyme (ME2) is well-known for its role in cell metabolism, which may be involved in cancer or epilepsy. We present potent ME2 inhibitors based on cyro-EM structures that target ME2 enzyme activity. Two structures of ME2-inhibitor complexes demonstrate that 5,5’-Methylenedisalicylic acid (MDSA) and embonic acid (EA) bind allosterically to ME2’s fumarate-binding site. Mutagenesis studies demonstrate that Asn35 and the Gln64-Tyr562 network are required for both inhibitors’ binding. ME2 overexpression increases pyruvate and NADH production while decreasing the cell’s NAD^+^/NADH ratio; however, ME2 knockdown has the opposite effect. MDSA and EA inhibit pyruvate synthesis and thus increase the NAD^+^/NADH ratio, implying that these two inhibitors interfere with metabolic changes by inhibiting cellular ME2 activity. ME2 silence or inhibiting ME2 activity with MDSA or EA decreases cellular respiration and ATP synthesis. Our findings suggest that ME2 is crucial for mitochondrial pyruvate and energy metabolism, as well as cellular respiration, and that ME2 inhibitors could be useful in the treatment of cancer or other diseases that involve these processes.

## Abstract (web summary)

Two specific inhibitors for emerging cancer therapy target malic enzyme 2 (ME2) are identified and are shown to bind to one of ME2 allosteric sites, resulting in inactive open conformation, inhibiting ME2-mediated cellular respiration and ATP production in the cell

## Introduction

Malic enzyme ME is a novel class of oxidative decarboxylases that catalyze the conversion of L-malate to pyruvate while simultaneously reducing NAD(P)^+^ to NAD(P)H^1–4^. Malic enzymes in mammals are classified into three isoforms, ME1, ME2, and ME3, based on their subcellular localization and cofactor specificity, with each serving a distinct physiological function. ME1 is a cytosolic NADP^+^-dependent ME involved in the generation of cytoplasmic NADPH for reductive biosynthesis and replenishment of the tricarboxylic acid (TCA) cycle intermediate by the reverse transformation of pyruvate to L-malate^5,6^. ME3 is a negligibly expressed mitochondrial NADP^+^-dependent ME that may be involved in the cycling of NADPH into the mitochondria^5^. ME2 is a NAD^+^ or NADP^+^-dependent ME found in mitochondria that is involved in the generation of mitochondrial NADH and NADPH^4,7–9^. ME2 is distinguished from the other two mammalian isoforms by its dual cofactor specificity and a complex allosteric regulatory system. Additionally, only the ME2 isoform cooperates with the substrate L-malate, and fumarate can allosterically activate the enzyme while ATP inhibits its enzymatic activity^10–19^.

ME2 was initially identified in hepatoma mitochondria^8^, and has since been identified in leukemia, melanoma, glioma, and breast cancer^9,20–22^ where it is strongly associated with cancer progression and survival. As a result, it has been identified as a promising target for cancer therapy^23^. It is also present in pancreatic islets of human, rat and mouse insulinoma cells, and it may contribute to amino acid-stimulated insulin secretion and provide sufficient pyruvate for increased Krebs cycle flux when glucose is limited^24,25^. ME2 is also required for the proliferation and differentiation of osteoblasts^26^. ME2 activity is extremely abundant in synaptic mitochondria in the brain, indicating that it plays a role in the pyruvate recycling pathway and in the maintenance of intramitochondrial reduced glutathione in synaptic terminals^27^. The ME2 gene has been linked to epilepsy syndromes. Using both case-control and family-based association methods, it was found to be associated with idiopathic generalized epilepsy (IGE) in one study.

ME2 has been identified with a gene associated with epilepsy syndromes. It was identified with the gene associated with idiopathic generalized epilepsy (IGE) in one study using both case-control and family-based association methods^28^. The haplotype of single-nucleotide polymorphism ME2 (SNP) has been linked to an increased risk of IGE and predisposes to adolescent-onset genetic generalized epilepsy^29^.

In tumor cells, glutaminolysis via the tricarboxylic acid cycle may cooperate with malate oxidation to pyruvate via ME2^4,9,30^. Glutamate and glutamine are used as energy sources in cancer cells, and ME2 may play a critical role in glutaminolysis^30,31^. ME2 converts L-malate, which is derived from glutamine, in the mitochondria to pyruvate and NAD(P)H^30–33^. By producing NADH and pyruvate, the ME2 may play an important role in energy production in rapidly proliferating tissues and tumor cells^8,31,34^; by producing NADPH, ME2 generates the reducing equivalents for glutathione reduction^35,36^.

ME2 has been shown to be negatively regulated by p53, and its expression protects cancer cells from the cellular senescence caused by p53^37^. Two functional response elements of p53 are located in the first intron of the ME2 gene, implying that p53 may act as a transcriptional repressor for ME2^37^. Indeed, a reciprocal regulatory relationship exists between p53 and malic enzymes, which determines the cell’s irreversible fate through the ME2-involved metabolic pathway^38^. We have demonstrated the importance of ME2 in cutaneous melanoma^20^. ME2 deficiency in melanoma cells results in decreased ATP levels and increased ROS levels, which triggers AMP-activated protein kinase (AMPK) activity, promoting p53 phosphorylation and activation, and ultimately cell death^20^. The effect of structural analogs of the malate substrate and the allosteric activator fumarate on human ME2 has been investigated^39,40^. Additionally, our laboratory discovered a small molecule inhibitor, embonic acid (EA), that is ME2-specific, and EA inhibits lung cancer cell growth and induces cellular senescence^41^.

Malic enzyme is a homotetramer, or dimer of dimers, in which the dimer interface is more tightly coupled than the tetramer interface is^42^. The crystal structures of human ME2 in complex with their ligands reveals that each monomer of the enzyme contains two additional ligand binding sites^11^. One site is located at the dimer interface and is responsible for binding the allosteric activator fumarate^11^. The other site, located at the tetramer interface, is capable of binding another nucleotide, such as NAD^+^ or ATP; this second nucleotide-binding site is referred to as the “exo site.” The nucleotide ligands in the exo site have discrete biological functions that include regulating the quaternary structure and catalysis of ME2^19^. Human ME2 can exist in both open and closed forms. The structure of human ME2 in its binary complex with the cofactor NAD^+^ is representative of open form I, whereas the structure of pentary complexes such as ME2-nucleotide (NAD^+^, NADH or ATP)-divalent cation (Mg^2+^ or Mn^2+^)-substrate (pyruvate or L-malate)-fumarate is representative of closed form II of the enzyme^10,11,43^. The human ME2 gene contains numerous single nucleotide variants (SNVs) in the coding region, which may have an effect on the enzyme’s function. We previously established that SNVs in the allosteric fumarate-binding site and the exo-site result in the inactivation or hyper-activation of ME2, and the resolved ME2-SNV structures provide a molecular basis for explaining the SNV enzymes’ abnormal kinetic properties^44^.

In this article, we describe the complex structures of ME2 and its allosteric inhibitors, demonstrating the inhibitors’ detrimental effect on the structure transition; we also discuss ME2’s role in energy metabolism. We investigated the efficacy of allosteric ME2 inhibitors, EA and MDSA, in inhibiting ME2-mediated pyruvate and energy metabolism in three non-cancerous cells, demonstrating ME2’s role in energy metabolism by increasing pyruvate and NADH production, which boosts ATP production, as well as in anti-oxidation when the mitochondria are subjected to a high level of oxidative stress. This article provides a perfect illustration of the allosteric regulation of enzymes, demonstrating the effects of allosteric inhibitors on their structure and function, as well as on cellular energy metabolism. Furthermore. using biochemical, biophysical, and cellular metabolism approaches, it explains the role of ME2 in the mitochondria, as well as the role of PFK in the cytoplasm; both play an energy-sensing role in their respective organelles.

## Results

### Disalicylic acid and naphthoic acid derivatives as potent inhibitors of human ME2

On the basis of site-directed mutagenesis of the dimer or tetramer interfaces, the fumarate-binding site, and the exo site, we previously reported that 4,4’-Methylene-bis(3-hydroxy-2-naphthoic acid), also known as embonic acid (EA), may act as an allosteric inhibitor of ME2^41^. Additionally, the inhibitory effects of a variety of disalicylic acid and naphthoic acid derivatives on ME2 enzymatic activity were investigated (Fig. S1). The chemical structures of these derivatives are listed in Table S1, along with their IC_50_ values. Among these compounds, a disalicylic acid, 5,5’-Methylenedisalicylic acid (MDSA) had a remarkable inhibitory effect on ME2 with an IC_50_ of 0.51 µM (Fig. S1a), while salicylic acid had a negligible inhibitory effect on ME2 (IC_50_ = 800.7 µM; Fig. S1b). 3-benzoylbenzoic acid, which has a carbon skeleton similar to MDSA, had a negligible inhibitory effect on ME2 activity, with an IC_50_ value of 652 µM, as shown in Fig. S1b.

EA, a bis 3-hydroxy-2-naphthoic acid, had a notable inhibitory effect on ME2 with an IC_50_ of 1.1 µM. (Fig. S1a). The inhibitory effect of 3-hydroxy-2-naphthoic acid with a hydroxyl group at 5’ position, 3,5-dihydroxy-2-naphthoic acid (IC_50_ of 12.4 µM; Fig. S1b) has a greater inhibitory effect than 3,5-dihydroxy-2-naphthoic acid with a hydroxyl or bromo group at 7’ position, 3,7-dihydroxy-2-naphthoic acid and 7-bromo-3-hydroxy-2-naphthoic acid (IC_50_ values of 37.6 µM and 307.8 µM, respectively; Fig. S1b). Naphthoic acid derivatives with adjacent hydroxyl groups, such as 1-hydroxy-2-naphthoic acid and 3-hydroxy-2-naphthoic acid, exhibit considerably less inhibitory effect on activity than EA (IC_50_ values of 74.7 µM and 105.4 µM, respectively; Fig. S1b). 2,6-dicarboxynaphthalene, a naphthalene containing dicarboxylic acid, had a negligible inhibitory effect on ME2 activity, with an IC_50_ value of 352.9 µM (Fig. S1b).

### Cryo-EM structures demonstrate that the ME2-specific inhibitor, MDSA or EA, binds to the enzyme at the dimer interface near the allosteric fumarate-binding site

In the active site of MEs, the residues necessary for substrate and metal binding are substantially conserved across ME1 and ME2^3,10,42,43^. ME2, but not ME1, is an allosteric enzyme, and fumarate or nucleotides bind to distinct allosteric sites to modulate ME2 activity^3,11,12,16,17,19^. In fact, unlike ME2, ME1 lacks an allosteric site at the dimer interface, so it cannot be activated by fumarate^3,15^. Sequence alignments and kinetic studies demonstrate this possibility^12,16,17,45^. The vast majority of allosteric site residues in ME2 are not conserved in ME1. Consequently, ME1 cannot be activated by fumarate, and it stands to reason that ME1 may not be sensitive to allosteric inhibitors.

**Fig. 1:**
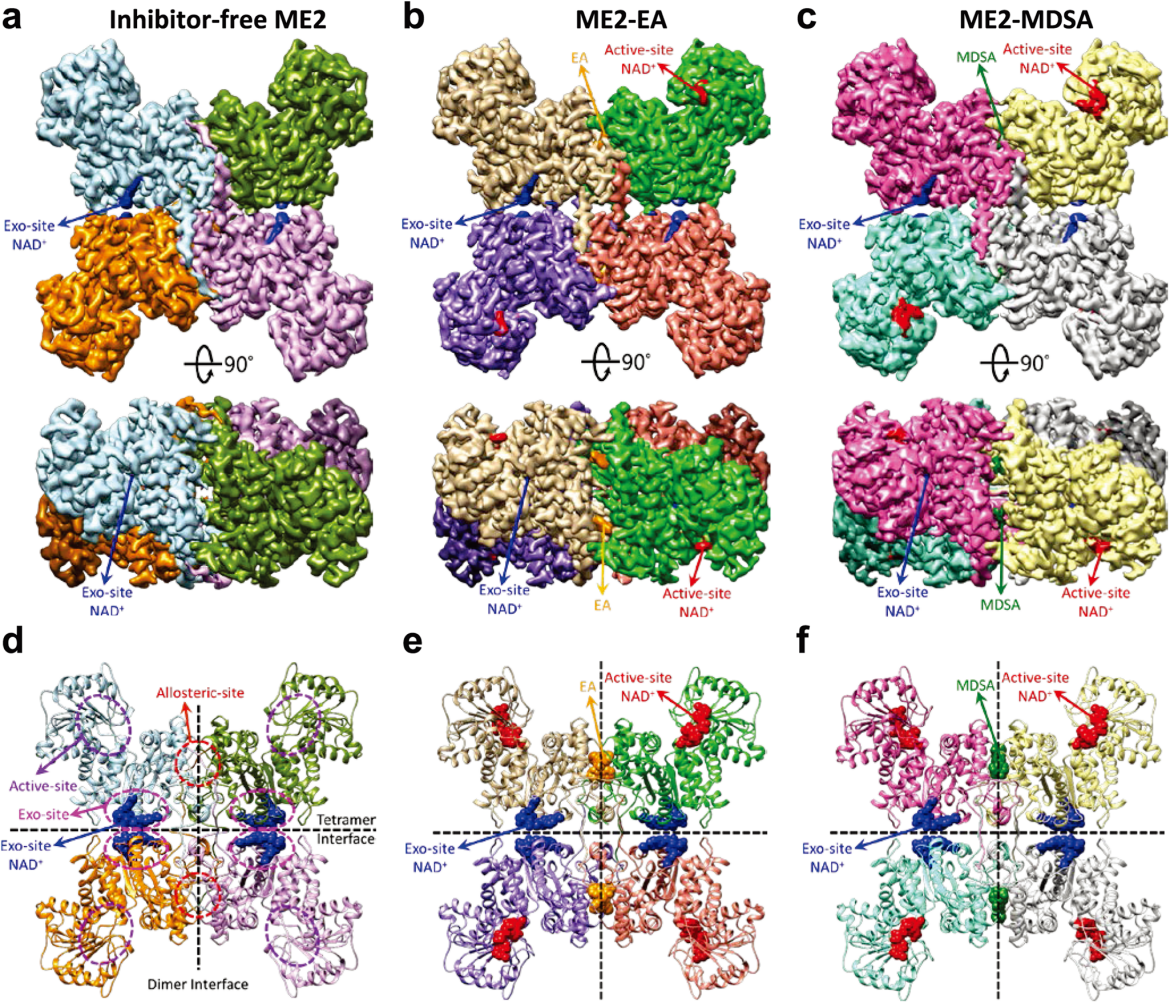
Cryo-EM structures of the ME2-NAD^+^ binary complex, and the ME2-NAD^+^-EA and ME2-NAD^+^-MDSA ternary complexes. **a** Cryo-EM structures of the ME2-NAD^+^ binary complex at ∼ 2.7 Å resolution. The exo-site NAD^+^ is highlighted in blue. The map is contoured at 6.7 σ above the mean. **b** Cryo-EM structure of the ME2-NAD^+^-EA ternary complex at ∼ 2.7 Å resolution. The inhibitor EA is highlighted in orange color. The active-site NAD^+^ is colored in red and exo-site NAD^+^ is colored in blue. The map is contoured at 8.3 σ. **c** Cryo-EM structure of the ME2-NAD^+^-MDSA ternary complex at ∼ 2.8 Å resolution. The inhibitor MDSA is depicted in green color. The active-site NAD^+^ is colored in red and exo-site NAD^+^ is colored in blue. The map is contoured at 8 σ. **d** Cartoon representations of the ME2 binary complex with exo-site NAD^+^ (blue spheres). The allosteric sites (indicated by red dashed circles), the active sites (indicated by purple dashed circles), and the exo sites (indicated by pink dashed circles) are demonstrated. Vertical and horizontal dashed lines denote the dimer and tetramer interfaces, respectively. **e** ME2 ternary complex with the allosteric-site EA (orange spheres), the active-site NAD^+^ (red spheres) and the exo-site NAD^+^ (blue spheres). **f** ME2 ternary complex with the allosteric-site MDSA (green spheres) and the active-site NAD^+^ (red spheres) and the exo-site NAD^+^ (blue spheres).

ME2 is highly sensitive to MDSA and EA inhibition, whereas ME1 is notably less sensitive, indicating that both inhibitors bind to allosteric sites. In this article, we demonstrate the cryo-EM structures of ME2-MDSA and ME2-EA revealing that the binding site for the ME2-specific inhibitors MDSA and EA is located at the dimer interface’s allosteric fumarate-binding site (Fig. 1). The structure reveals that Asp37 in ME1 may sterically hinder the binding of MDSA and EA whereas glycine is the corresponding residue in ME2.

The cryo-EM structure of human ME2 in ternary complexes with NAD and inhibitors (EA or MDSA) were determined at 2.72 Å and 2.82 Å resolutions, respectively (Fig. S2 and Table S2). Additionally, we determined the cryo-EM structure of human ME2 in the absence of the inhibitor to a resolution of 2.72 Å (Fig. S2 and Table S2). The inhibitor-free ME2, EA-ME2, and MDSA-ME2 single-particle cryo-EM analyses were displayed as a representative cryo-EM image, reference-free 2D class averages, resolution maps for the final reconstructions, gold standard FSC plots for the 3D reconstructions, and euler angle distribution of the particle images (Fig. S2a–e, respectively). The data processing workflows for inhibitor-free-ME2 structure, ME2-EA, and ME2-MDSA are depicted in Figs. S3–S5.

The overall cryo-EM structure of tetrameric inhibitor-free-ME2 (Figs. 1a and 1d) are comparable to that of the binary complex with NAD^+^^42^. As shown in the two-dimensional class average of electron microscope images (Fig. S2b), the four monomers are positioned in the structure’s four corners. In the cryo-EM structure of human ME2 without the inhibitor, the sample was purified in the absence of the cofactors NAD^+^, and only one NAD^+^ molecule is observed in the exo site of the ME2 subunit (Figs. 1a and 1d). The active site of inhibitor-free structure is exposed to the solvent, similar to the open form of ME2^42^.

Both ME2-inhibitor complexes (ME2-EA and ME2-MDSA) were generated in the presence of the cofactors NAD^+^ and Mg^2+^, the natural substrate pyruvate (PYR), and the inhibitor (EA or MDSA), but the structures demonstrate that only the inhibitor (EA or MDSA) binds to the allosteric regulatory site at the dimer interface (Figs. 1b, c, respectively), while NAD^+^ molecules appear in each active site and exo-site at the tetramer interface (Figs. 1e, f). The nicotinamide mononucleotide (NMN) part of NAD^+^ has considerably lower electron densities in these three cryo-EM structures of human ME2 (Fig. 1), implying that it may be highly disordered. The poorly resolved NMN component of NAD^+^ is also seen in the crystal structure of ME2^11^.

Previous structural studies have uncovered the open and closed conformations of ME2, which correspond to the enzyme’s inactive and active states, respectively. The active site is completely exposed to the solvent in the inactive open form. After the binding of divalent cations (Mn^2+^ or Mg^2+^) and substrates (malate or pyruvate), active site closure is predominantly mediated by the rigid-body movement of the active site domain toward the allosteric site. As a consequence, divalent cations and substrates are protected from the solvent in the active closed form^3,44^.

In the structures containing bound inhibitors (ME2-EA and ME2-MDSA), both EA and MDSA are bound to the allosteric site at the dimer interface. Unlike the fumarate, the bulkiness of both inhibitors necessitates additional space, thus pushing domain B toward the active site. This movement alters the spatial locations of Glu255, Asp256, and Asp279. Arg165 specifically replaces Asp256 at the side chain position. It has been demonstrated that the highly conserved amino acids Glu255, Asp256, and Asp279 are required for catalysis and chelation of divalent ions in the active site of MEs^3,10,11,43^. Therefore, the spatial arrangements induced by EA and MDSA lead to the loss of divalent ion at the active site and inhibit catalysis.

### Allosteric site coordination in ME2-EA and ME2-MDSA ternary complexes

**Fig. 2:**
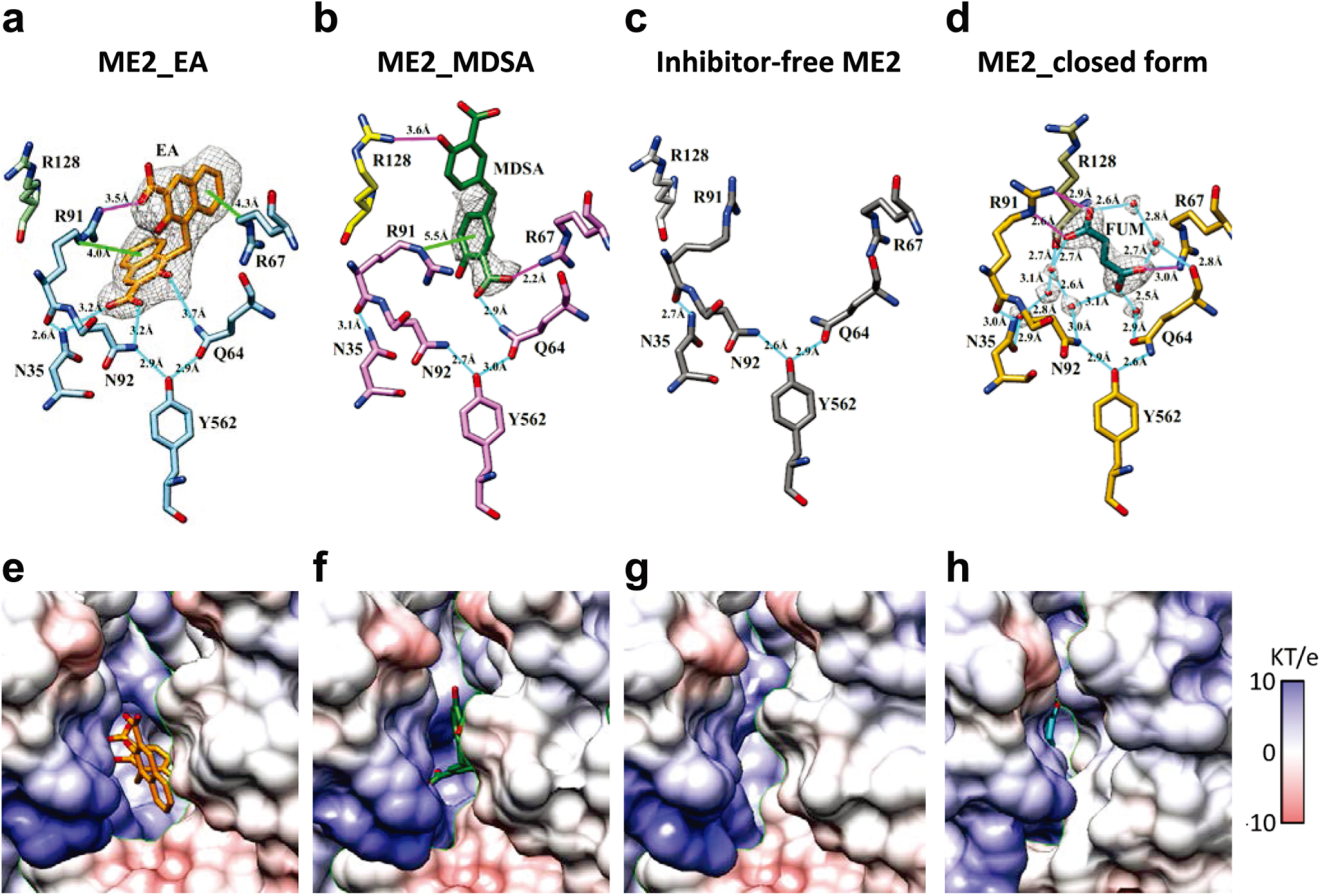
Allosteric site coordination of ME2-EA, ME2-MDSA, inhibitor-free ME2, and closed forms of ME2. The structures illustrate the allosteric site’s ligand interactions (upper panels) and the Coulombic surfaces surrounding the allosteric site (lower panels). The gray meshes represent the densities of ligands, and the sticks represent the interacting residues. Hydrogen bonding interactions are indicated by cyan lines, cation-π interactions by green lines, and ion pairs by magenta lines. **a**, **e** The inhibitor EA is depicted in orange sticks in the ME2-EA complex. The map is contoured at 6.5 σ above the mean. **b**, **f** MDSA is depicted in green sticks in the ME2-MDSA complex. The map is contoured at 7.5 σ. **c**, **g** Allosteric site of the inhibitor-free ME2. **d**, **h** The activator fumarate is depicted in dark cyan sticks in the ME2 closed form (PDB ID: 1PJ3). The map is contoured at 1.8 σ. Coulombic surfaces were calculated using the default settings in UCSF Chimera^55^.

Fumarate can trigger the catalytic activity of human ME2, and the allosteric activator has been found at the dimer interface (Fig. 2d), approximately 30 Å distant from each active site (Fig. 1). The activator interacts with the side chains of Arg67 and Arg91 in the allosteric region (Fig. 2d), and mutagenesis studies confirm the significance of the Arg67 and Arg91 residues in fumarate binding^11,43^.

The cryo-EM structure of the ME2-inhibitor complex (ME2-EA and ME2-MDSA) reveals the inhibitor EA or MDSA at the dimer interface’s allosteric regulatory region (Figs. 1b, c, respectively). EA interacts with ME2 via ion pairings, cation-pi interactions, hydrogen bonding, and hydrophobic interactions with surrounding amino acids (Fig. 2a). The residues, Arg67 and Arg91 have cation-π interactions with EA, while Arg91 additionally forms an ion pair with EA. Asn35, Asn92, and Gln64 interact with EA via hydrogen bonds, and hydrogen networks exist between the Gln64, Asn92, and Tyr562 side chains (Fig. 2a).

MDSA forms ion pairs with adjacent subunit residues Arg67 and Arg128, whereas Arg91 interacts with MDSA via a cation-π interaction. Gln64 forms hydrogen bonds with MDSA, and hydrogen networks also exist between the Gln64, Asn92, and Tyr562 side chains (Fig. 2b). In comparison to the ME2 structure without the inhibitor (Fig. 2c), Arg91 from the ME2-EA complex shifts slightly toward EA, forming a cation-pi interaction (Fig. 2a). In the structure of the ME2-MDSA complex, not only does the side chain of Arg91 change direction toward MDSA, but the side chain of Arg128 from another subunit is also moved and interacts with it (Fig. 2b). Unlike fumarate, which primarily interacts with the side chains of Arg67 and Arg91 via ion-pair interactions (Fig. 2d), the inhibitors EA and MDSA primarily interact with the side chains of Arg67 and Arg91 via ion-pair and cation-pi interactions (Figs. 2a, b). The allosteric site pocket is extensible, allowing it to accommodate molecules larger than fumarate, such as EA and MDSA (Figs. 2e–h).

### Active-site NAD^+^ coordination in ME2-EA and ME2-MDSA ternary complexes

**Fig. 3:**
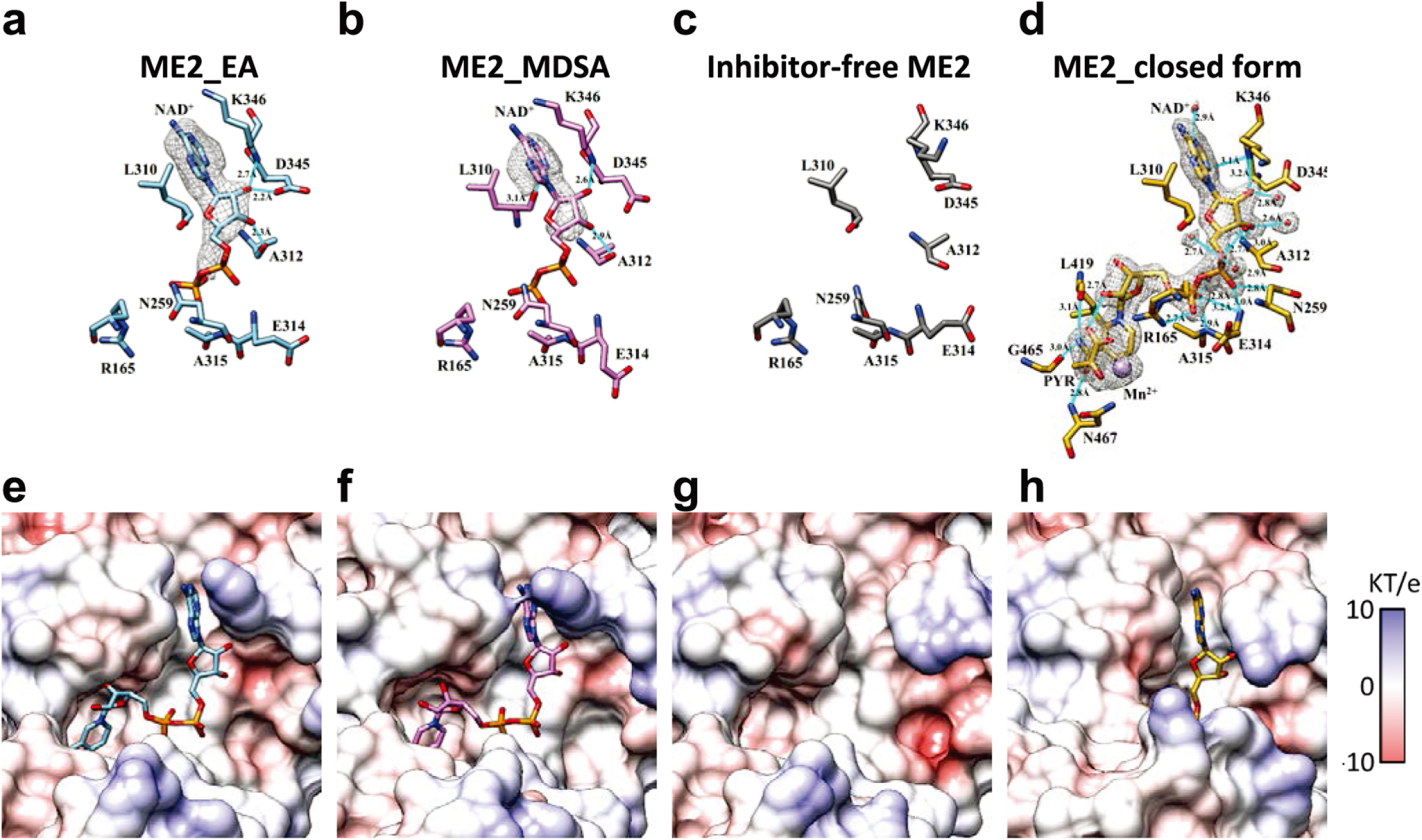
Active site NAD^+^ coordination of ME2_EA, ME2_MDSA, inhibitor-free ME2, and closed forms of ME2. The structures illustrate the ligand interactions of active-site NAD^+^ (**a**, **b**, **c**, and **d**) and the Coulombic surfaces surrounding the active site (**e**, **f**, **g**, and **h**). The gray meshes represent the densities of ligands, and the sticks represent the interacting residues. Hydrogen bonding interactions are indicated by cyan lines, cation-π interactions by green lines, and ion pairs by magenta lines. In upper panels, only the ADP portion of the NAD^+^ molecule is shown. **a**, **e** The active-site NAD^+^ and the interacting residues are depicted as light blue sticks in the ME2-EA complex. The map is contoured at 5 σ above the mean. **b**, **f** The active-site NAD^+^ and the interacting residues are depicted as pink sticks in the ME2-MDSA complex. The map is contoured at 7.5 σ above the mean. **c**, **g** The NAD^+^ was not observed in the active site, and the residues surrounding the pocket in the inhibitor-free ME2 are depicted as gray sticks. **d**, **h** The active-site NAD^+^ and the interacting residues are depicted as yellow sticks in the ME2 closed form (PDB ID: 1PJ3). The map is contoured at 1.5 σ above the mean. Coulombic surfaces were calculated using the default settings in UCSF Chimera^55^.

The four active sites are located in the corners of the human ME2 tetramer structure, approximately 60 Å apart (Fig. 1). The cryo-EM structure of ME2 open form without an inhibitor is a binary complex with only the exo-site NAD^+^; the active sites have no ligands and are completely exposed to the solvent. (Fig. 3c). The closed form of ME2 is a pentary complex with natural product pyruvate, cofactor NAD^+^, Mn^2+^, and fumarate^3,43^. Despite the addition of pyruvate during cryo-EM preparation of both samples (Figs. 1b, c, respectively), the substrate pyruvate was not detected in the ME2-EA and ME2-MDSA ternary complex structures, and the active site contained only the NAD^+^ molecule (Figs. 3a, b).

The substrate pyruvate, cofactor NAD^+^, and divalent cations Mg^2+^ are all bound to the surrounding residues in the closed form of ME2’s active site (Fig. 3d), and they are shielded in the deep cleft (Fig. 3h). Comparing the active sites in the structures of ME2-EA and ME2-MDSA and in the open and closed forms of ME2, the active site pockets of ME2-EA (Fig. 3a) and ME2-MDSA (Fig. 3b) are in an open state similar to the open form of ME2 (Fig. 3c), indicating that the binding of EA and MDSA locks enzyme conformation in the catalytically inactive open form. The active site NAD^+^ pocket is slightly open, which may cause NMN moiety more flexible (Figs. 3e–h).

### Exo-site NAD^+^ coordination in ME2-EA and ME2-MDSA ternary complexes

The human ME2 exo site in each subunit is located at the tetramer interface, approximately 35 Å distant from the active site (Fig. 1). Our cryo-EM structures of the ME2-EA and ME2-MDSA ternary complexes, as well as the open form of ME2, reveal that only the ADP portion of the NAD^+^ molecule is ordered in the exo sites of ME2 (Fig. S6), consistent with previous crystallographic observations (PDB ID: 1PJ3, Fig. S6d). NAD^+^‘s adenine base forms hydrogen bonds with the amide of Arg194 and the carbonyl of Arg556, while the side chains of Arg197 form hydrogen bonds with NAD^+^‘s ribose. Hydrogen bonds are formed between the phosphate groups of NAD^+^ and the side chains of Arg542 and Arg556 (Figs. S6a, S6b and S6c). A comparison of the ligand interactions of exo-site NAD^+^ in the structures of ME2-EA and ME2-MDSA and the open and closed forms of ME2 reveals that the ADP portions are oriented similarly in them (Fig. S6).

### Inhibition of ME2 inhibitor-binding site mutants with MDSA or EA

Based on the structures of ME2-EA and ME2-MDSA, we designed 16 ME2 mutants and assessed their susceptibility to MDSA or EA inhibition to determine which amino acid residues are required for MDSA or EA binding and inhibition (Fig. S7). Arg67 and Arg91 are fumarate’s direct ligands; both R67A and R91A mutants were less sensitive to MDSA or EA, with increased IC_50_ values (Table 1). Because Arg67 is the ligand that directly interacts with MDSA, the R67A mutant demonstrated activity that was notably less inhibited by MDSA, with an IC_50_ value of 185 µM, which was over 300-fold that of the WT (Table 1). Arg67 and Glu59 form a salt bridge, and Glu59 is ion-paired with Lys57. However, the K57 and E59 mutants remained susceptible to MDSA or EA inhibition, with IC_50_ values comparable to those of the WT (Table 1).

**Table 1:** The IC_50_ values of MDSA and EA as inhibitors of human wild-type and mutant ME2.

ME2	IC_50,MDSA_ (µM)	IC_50,EA_ (µM)
WT	0.51 ± 0.03	1.14 ± 0.05
N35A	237.8 ± 24.5	84.0 ± 4.6
N35D	20.1 ± 2.0	10.1 ± 1.1
N35Q	510.5 ± 29.2	189.7 ± 4.8
K57A	0.57 ± 0.07	0.31 ± 0.03
K57S	0.26 ± 0.03	0.69 ± 0.09
E59A	0.25 ± 0.08	1.93 ± 0.18
E59N	0.50 ± 0.05	3.32 ± 0.19
Q64A	18.0 ± 1.8	8.27 ± 1.06
Q64E	91.8 ± 4.6	41.2 ± 3.7
Q64N	62.4 ± 5.4	38.2 ± 4.4
R67A	184.5 ± 9.45	15.14 ± 1.66
R91A	2.89 ± 0.23	8.13 ± 0.56
N92A	3.53 ± 0.72	6.66 ± 0.37
N92Q	1.28 ± 0.17	1.77 ± 0.13
R128A	2.30 ± 0.32	5.25 ± 0.38
Y562A	40.8 ± 5.0	46.1 ± 2.5

Gln64 serves as the primary ligand for MDSA or EA binding, while Tyr562 forms a hydrogen bond with Gln64. Q64N had IC_50_ values of 62.4 µM and 38.2 µM, and Y562A had IC_50_ values of 40.8 µM and 46.1 µM, for MDSA and EA, respectively, values which were obviously greater than those for WT (Table 1), indicating that the Gln64-Tyr562 pair is required for MDSA or EA binding in the allosteric site.

Due to the direct binding of Asn35 to EA, the IC_50_ value of N35D was 10-fold greater than that of WT, whereas the IC_50_ values of N35A and N35Q were 70- and 160-fold greater than those of WT (Table 1), indicating that the structural specificity of the side-chain of Asn35 is crucial for EA binding. Consequently, despite the fact that Asn35 does not interact directly with MDSA, the N35 mutants were less susceptible to MDSA inhibition due to the side-chain effect of Asn35. Asn92 has a direct interaction with EA, while Arg128 has a direct interaction with MDSA. Both residues are not required for MDSA or EA binding, as indicated by the fact that the IC_50_ values of the N92A, N92Q, and R128A mutants were not markedly increased, indicating that these mutants remained susceptible to MDSA or EA inhibition (Table 1).

While these inhibitor-binding mutants of ME2 exhibit a wide range of kinetic properties (Table S3) and the majority are insensitive to the allosteric activator fumarate (Fig. S8), as previously reported in the literature^44^, their overall secondary structures are quite similar to those of the WT (Fig. S9), indicating that the mutation of these residues did not result in substantial conformational changes, and the susceptibility of the ME2 mutants to MDSA or EA inhibition was determined by the local geometry of the allosteric site of ME2.

### MDSA or EA has a pronounced effect on the ME2-associated metabolic pathway

MDSA and EA were introduced to determine their inhibitory effect on cellular ME2 in three non-cancerous cell lines: HEK293T (human embryonic kidney 293 cells), HFL-1 (human lung fibroblast cells), and MRC-5 (human embryonal lung fibroblast cells), and two cancerous cell lines: H1299 (human non-small cell lung carcinoma), and MCF-7 (human breast adenocarcinoma). All five cell lines exhibited ME2 expression (Figs. S10a–10d). We also established stable HEK293T cells with ME2 overexpression plasmids (pcDNA3-vector and pcDNA3-ME2; Fig. S10e) and with shRNA for ME2 knockdown (shCon and shME2; Fig. S10a), as a positive and negative controls, respectively.

**Fig. 4:**
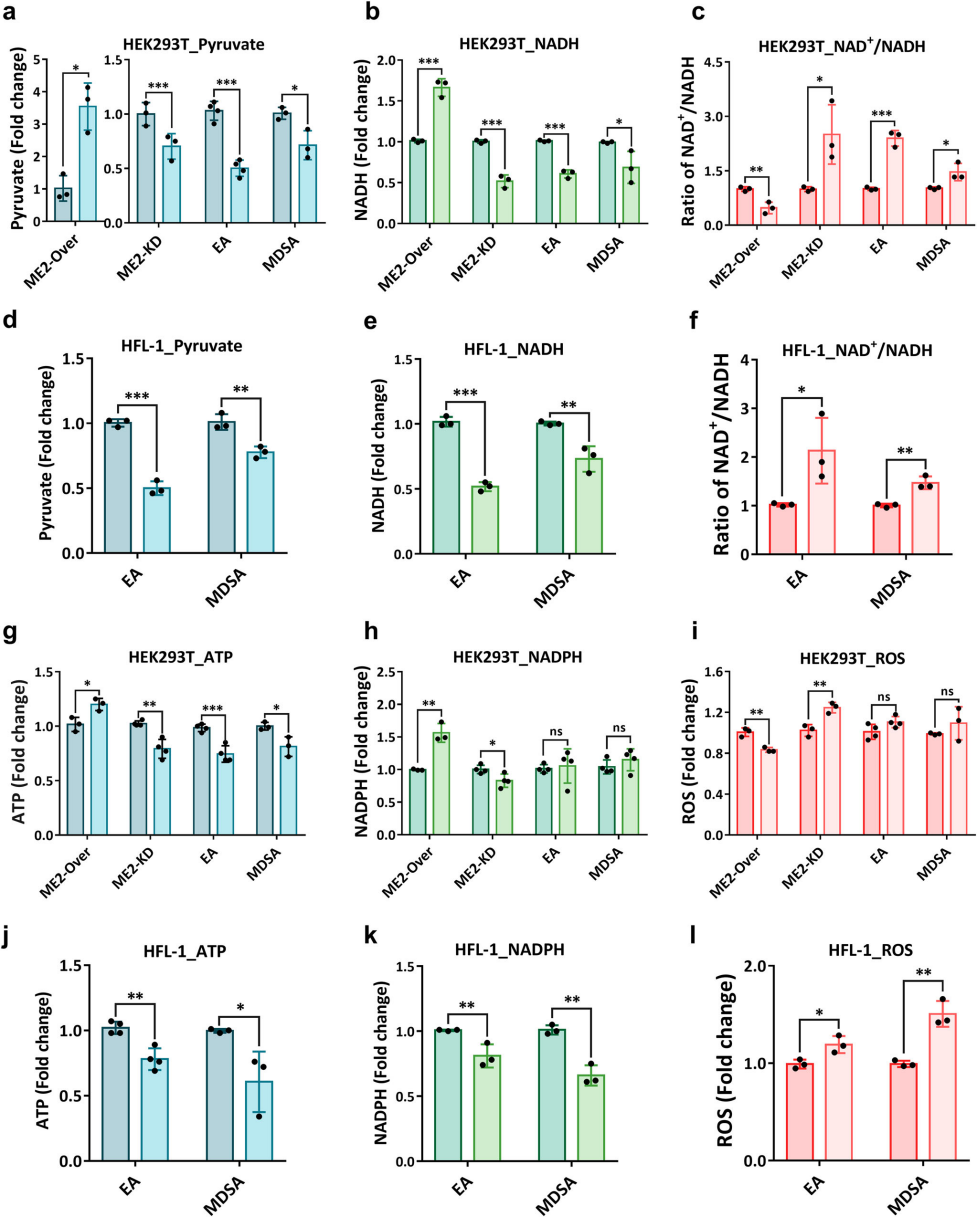
Change in the levels of pyruvate and NADH, the ratio of NAD^+^/NADH, ATP, NADPH, and reactive oxygen species (ROS) in HEK293T and HFL-1 cells treated with EA or MDSA. The fold change in cellular pyruvate and NADH levels, the NAD^+^/NADH ratio, ATP, NADPH, and reactive oxygen species (ROS) in ME2-overexpressing (ME2-Over), ME2-knockdown (ME2-KD), EA-treated, and MDSA-treated cells. **a** The fold change in pyruvate levels in HEK293T cells. *N* = 3-4. Unpaired Student’s *t*-test. ^*^p < 0.05, ^***^p < 0.001. **b** The fold change NADH levels in HEK293T cells. *N* = 3. Unpaired Student’s *t*-test. ^*^p < 0.05, ^***^p < 0.001. **c** The ratio of NAD^+^/NADH in HEK293T cells. *N* = 3. Unpaired Student’s *t*-test. ^*^p < 0.05, ^**^p < 0.01, ^***^p < 0.001. **d** The fold change in pyruvate levels in HFL-1 cells. *N* = 3. Unpaired Student’s *t*-test. ^**^p < 0.01, ^***^p < 0.001. **e** The fold change in NADH levels in HFL-1 cells. *N* = 3. Unpaired Student’s *t*-test. ^**^p < 0.01, ^***^p < 0.001. **f** The fold change in the ratio of NAD^+^/NADH in HFL-1 cells. *N* = 3. Unpaired Student’s *t*-test. ^*^p < 0.05, ^**^p < 0.01. **g** The fold change in ATP levels in HEK293T cells. *N* = 3-4. Unpaired Student’s *t*-test. ^*^p < 0.05, ^**^p < 0.01, ^***^p < 0.001. **h** The fold change in NADPH levels in HEK293T cells. *N* = 3-4. Unpaired Student’s *t*-test. ^**^p < 0.01. ns, no statistical significance. **i** The fold change in ROS levels in HEK293T cells. *N* = 3-4. Unpaired Student’s *t*-test. ^**^p < 0.01. ns, no statistical significance. **j** The fold change in ATP levels in HFL-1 cells. *N* = 3-4. Unpaired Student’s *t*-test. ^*^p < 0.05, ^**^p < 0.01. **k** The fold change in NADPH levels in HEK293T cells. *N* = 3. Unpaired Student’s *t*-test. ^**^p < 0.01. **l** The fold change in ROS levels in HEK293T cells. *N* = 3. Unpaired Student’s *t*-test. ^*^p < 0.05, ^**^p < 0.01. The bar graphs illustrate the fold change in the levels of these metabolites after 48 h. Error bars are mean ± SD.

ME2 catalyzes the oxidation of malate followed by the reduction of NAD^+^ or NADP^+^ to form pyruvate and NADH or NADPH. As a result, the levels of pyruvate, NADH, and NADPH, as well as the NAD^+^/NADH ratio were determined first in ME2-overexpressing and ME2-knockdown HEK293T cells (Fig. 4). ME2-overexpressing cells had increased pyruvate and NADH levels, and a decreased NAD^+^/NADH ratio (Figs. 4a, b, c, respectively), whereas ME2-silenced cells had decreased pyruvate and NADH levels, and an increased NAD^+^/NADH ratio (Figs. 4a, b, c, respectively). When treated with MDSA or EA, HEK293T, HFL-1, and MRC-5 cells were also susceptible to ME2 inhibition. EA or MDSA treatment effectively inhibited ME2 activity in HEK293T, HFL-1, and MRC-5 cells, resulting in a decrease in pyruvate (Figs. 4a, d, and S11a, respectively) and NADH levels (Figs. 4b, e, and S11b, respectively) and an increase in the NAD^+^/NADH ratio (Figs. 4c, f, and S11c, respectively), similar to those of ME2-silence (Figs. 4a–c).

The NAD^+^/NADH ratio and ATP levels in the cell are critical indicators of energy metabolism. ME2-overexpressing cells had a lower NAD^+^/NADH ratio (Fig. 4c) and higher ATP levels (Fig. 4g), whereas ME2-silenced cells experienced the opposite (Figs. 4c, g), indicating that ME2 is positively correlated with energy metabolism. EA or MDSA treatment increased the NAD^+^/NADH ratio and decreased ATP levels in HEK293T cells (Figs. 4c, g, respectively). Similar results were observed in EA or MDSA-treated HFL-1 and MRC-5 cells. EA or MDSA treatment in HEK293T, HFL-1, and MRC-5 cells resulted in a decrease in ATP (Fig. 4g, j, and S11d, respectively) and NADH levels (Figs. 4b, e, and S11b, respectively), indicating that MDSA and EA were able to negatively regulate the cell’s energy metabolism by inhibiting ME2 activity.

The levels of reactive oxygen species (ROS) in HEK293T cells treated with MDSA or EA remained constant (Fig. 4i), which corresponded to unchanged NADPH levels (Fig. 4h). In MRC-5 cells, EA treatment resulted in a decrease in NADPH production (Fig. S11e), which resulted in an increase in ROS level (Fig. S11f). After being treated with MDSA or EA, HFL-1 cells also produced less NADPH (Fig. 4k), while production of ROS increased at the same time (Fig. 4l). It is possible that the oxidative stress caused by increased ROS and decreased NADPH in HFL-1 cells is the cause of the greatest effect of MDSA or EA treatment on cell viability seen with the HFL-1 cell (Fig.S12c). The HFL-1 cell was the most susceptible of the three non-cancerous cells to MDSA- or EA-mediated ME2 inhibition (Fig. S12a–S12c); this was due to the fact that MDSA and EA had a pronounced suppressive effect on it, reducing not only pyruvate and NADH levels (Figs. 4d and 4e, respectively), but also NADPH levels (Fig. 4k).

**Fig. 5:**
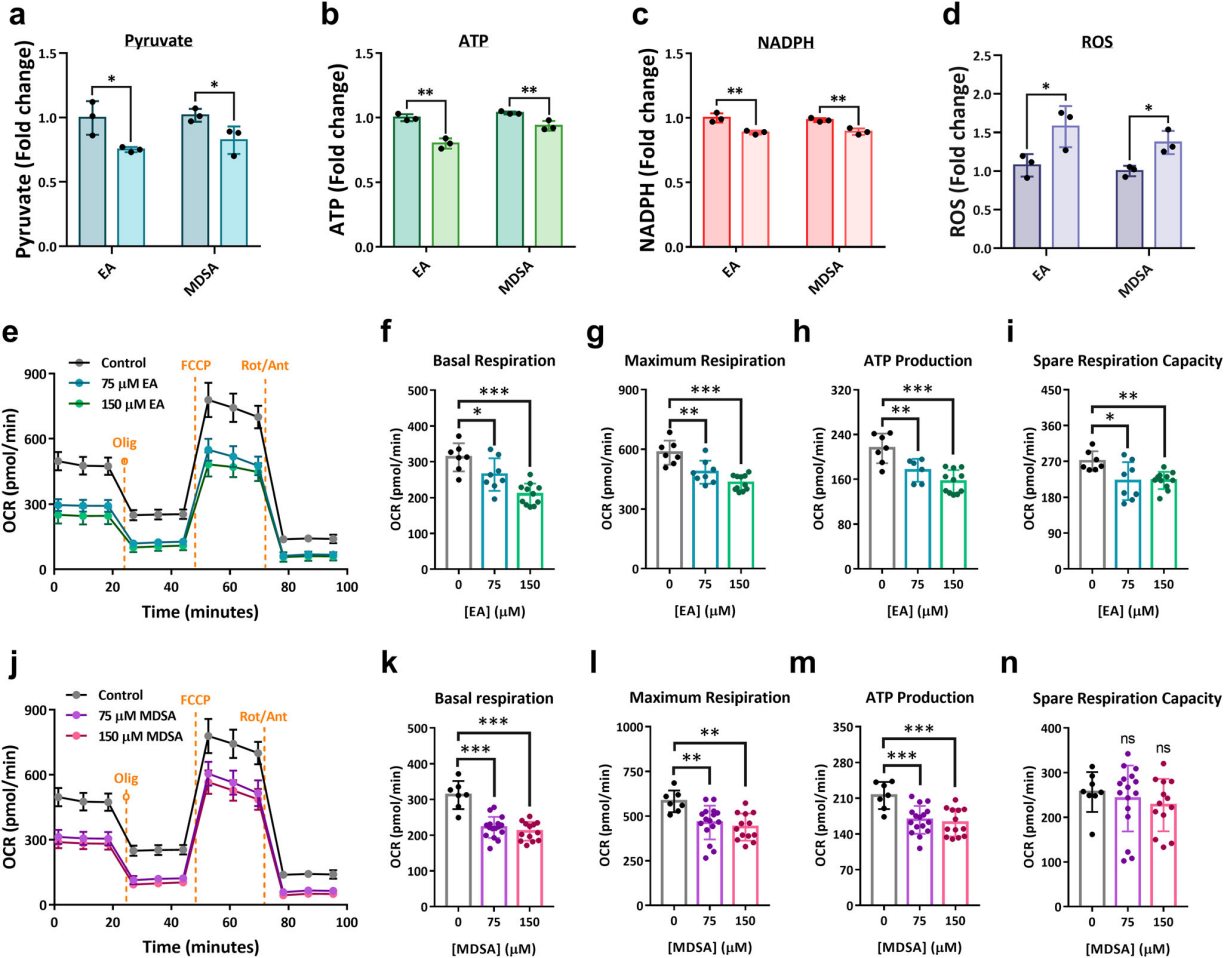
Changes in the levels of pyruvate, ATP, NADPH, ROS, and oxygen consumption rate (OCR) in H1299 cells treated with EA or MDSA. The proportional change of cellular pyruvate, ATP, NADPH, and ROS in H1299 cells in the presence of EA or MDSA (0, 75, and 150 µM). **a** The fold change in pyruvate levels. *N* = 3. Unpaired Student’s *t*-test. ^*^p < 0.05. **b** The fold change in ATP levels. *N* = 3. Unpaired Student’s *t*-test. ^**^p < 0.01. **c** The fold change in NADPH levels. *N* = 3. Unpaired Student’s *t*-test. ^**^p < 0.01. **d** The fold change in ROS levels. *N* = 3. Unpaired Student’s *t*-test. ^*^p < 0.05. **e** The oxygen consumption rate in EA-treated H1299 cells. *N* = 3. **f** The basal respiration rate. *N* = 7, 8 and 11, from left to right. One-way ANOVA with Dunnett’s test. ^*^p < 0.05, ^***^p < 0.001. **g** The maximal respiration rate. *N* = 7, 8 and 11, from left to right. One-way ANOVA with Dunnett’s test. ^**^p < 0.01, ^***^p < 0.001. **h** ATP production. *N* = 7, 6 and 11, from left to right. One-way ANOVA with Dunnett’s test. ^*^p < 0.05, ^***^p < 0.001. **i** The spare respiration capacity. *N* = 7, 8 and 11, from left to right. One-way ANOVA with Dunnett’s test. ^*^p < 0.05, ^***^p < 0.001. **j** The oxygen consumption rate in MDSA-treated H1299 cells (*N* = 3–5). **k** The basal respiration rate. *N* = 7, 16 and 13, from left to right. One-way ANOVA with Dunnett’s test. ^***^p < 0.001. **l** The maximal respiration rate. *N* = 7, 16 and 13, from left to right. One-way ANOVA with Dunnett’s test. ^*^p < 0.05, ^***^p < 0.001. **m** ATP production. *N* = 7, 16 and 13, from left to right. One-way ANOVA with Dunnett’s test. ^***^p < 0.001. **n** The spare res*p*iration capacity. *N* = 8, 16 and 13, from left to right. One-way ANOVA with Dunnett’s test. ns, no statistical significance. Error bars are mean ± SD. Oligo: Oligomycin; FCCP: Carbonyl cyanide-4 (trifluoromethoxy) phenylhydrazone; Rot/Ant: Rotenone/Antimycin A.

We continued to examine the efficacy of EA or MDSA on ME2-associated metabolic alterations in H1299 and MCF-7 cancerous cell lines (Fig. 5 and S13, respectively). ME2 was found in both cell lines (Fig. S10d). It was intriguing that H1299 cells were sensitive to EA or MDSA, as evidenced by a reduction in pyruvate, ATP, and NADPH production and an increase in ROS production (Figs. 5a–d, respectively). An increase in ROS and a decrease in NADPH in H1299 cells resulted in obvious cell mortality (Fig. S12d). In contrast, neither EA nor MDSA has substantial effects on ME2-directed metabolism in MCF-7 cells (Fig. S13a–S13d) or cell viability in MCF-7 cells (Fig. S12e), indicating that the effect of the drug on ME2-expressed cells is variable.

Mass spectrometry was used to determine the changes in pyruvate, malate, phosphoenolpyruvate (PEP), and glucose levels following MDSA or EA treatment (Fig. S14). The inhibition of ME2 by MDSA or EA, as anticipated, resulted in a decrease in pyruvate (Fig. 4 and S14). On the other hand, the level of cellular malate remained relatively constant, indicating that inhibiting ME2 activity does not result in malate accumulation (Fig. S14). Additionally, inhibiting ME2 activity had no effect on the cellular levels of PEP and glucose (Fig. S14). As a result of these findings, it can be concluded that ME2 inhibition decreased pyruvate generation without impairing glycolysis.

### MDSA or EA-mediated ME2 inhibition reduces cellular respiration and ATP production

**Fig. 6:**
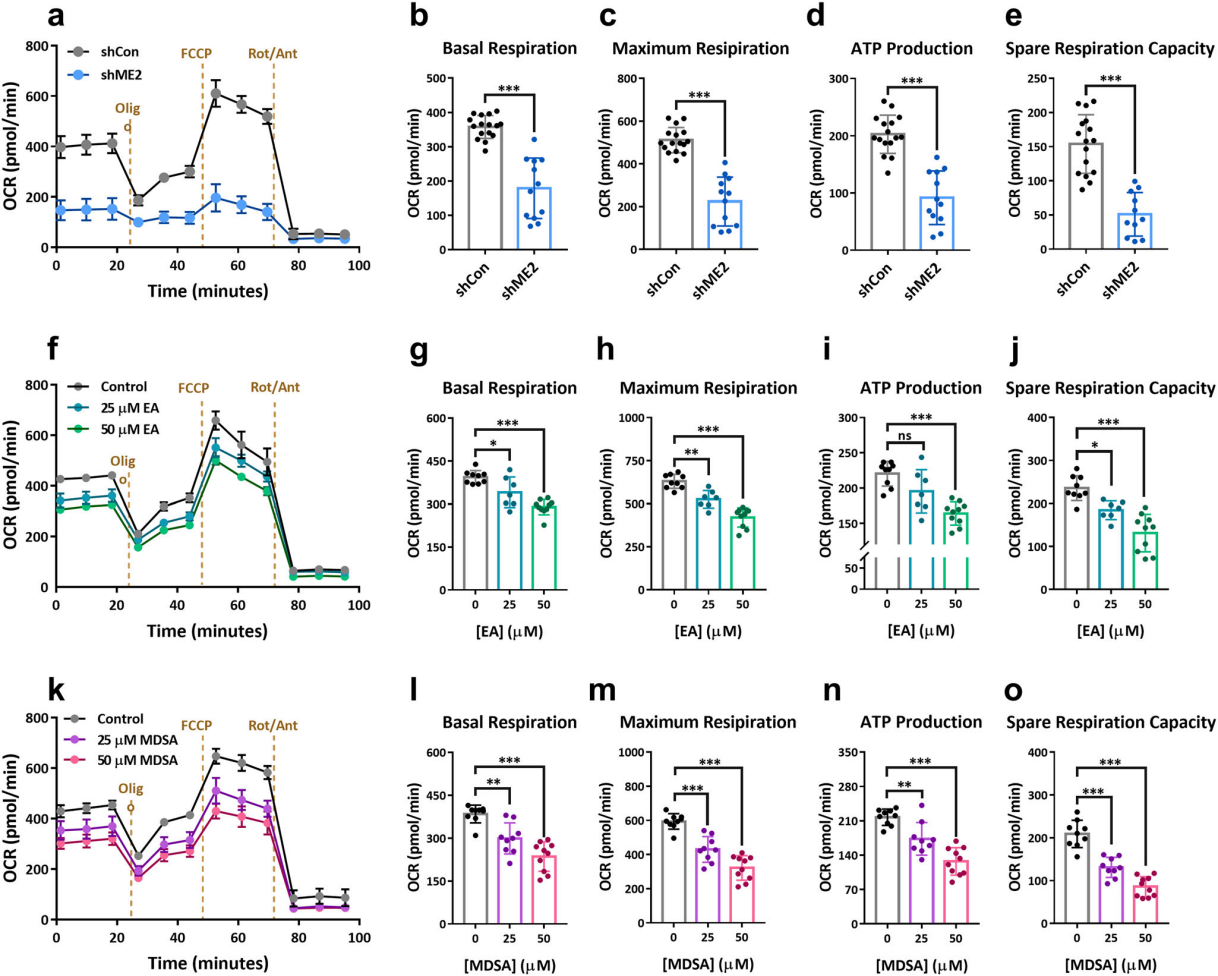
Fold change in oxygen consumption rate (OCR) of HEK293T cells in the presence of allosteric inhibitors EA or MDSA. **a** The oxygen consumption rate in ME2-control (shCon) and ME2-knockdown (shME2) HEK293T cells. *N* = 3–6. **b** The basal respiration rate. *N* = 16 and 12, from left to right. Unpaired Student’s *t*-test. ^***^p < 0.001. **c** The maximal respiration rate. *N* = 16 and 12, from left to right. Unpaired Student’s *t*-test. ^***^p < 0.001. **d** ATP production. *N* = 16 and 13, from left to right. Unpaired Student’s *t*-test. ^***^p < 0.001. **e** The spare respiration capacity. *N* = 16 and 11, from left to right. Unpaired Student’s *t*-test. ^**^p < 0.01. **f** The oxygen consumption rate in EA-treated HEK293T cells. N = 3-4. **g** The basal respiration rate. N = 9, 7 and 10, from left to right. One-way ANOVA with Dunnett’s test. ^*^p < 0.05, ^***^p < 0.001. **h** The maximal respiration rate. *N* = 9, 7 and 10, from left to right. One-way ANOVA with Dunnett’s test. ^**^p < 0.01, ^***^p < 0.001. **i** ATP production. *N* = 9, 7 and 10, from left to right. One-way ANOVA with Dunnett’s test. ^***^p < 0.001. ns, no statistical significance. **j** The spare respiration capacity. *N* = 9, 7 and 10, from left to right. One-way ANOVA with Dunnett’s test. ^*^p < 0.05, ^***^p < 0.001. **k** The oxygen consumption rate in MDSA-treated HEK293T cells. *N* = 3-5. **l** The basal respiration rate. *N* = 10, 9 and 10, from left to right. One-way ANOVA with Dunnett’s test. ^**^p < 0.01, ^***^p < 0.001. **m** The maximal respiration rate. *N* = 9, 9 and 10, from left to right. One-way ANOVA with Dunnett’s test. ^***^p < 0.001. **n** ATP production. *N* = 9, 9 and 10, from left to right. One-way ANOVA with Dunnett’s test. ^**^p < 0.01, ^***^p < 0.001. **o** The spare respiration capacity. *N* = 9, 9 and 10, from left to right. One-way ANOVA with Dunnett’s test. ^***^p < 0.001. Error bars are mean ± SD. Oligo Oligomycin, FCCP Carb**o**nyl cyanide-4 (trifluoromethoxy) phenylhydrazone, Rot/Ant Rotenone/Antimycin A.

We conducted oxygen consumption rate (OCR) experiments to determine the respiration capacity and the amount of ATP produced by oxidative phosphorylation (Fig. 6). The Agilent Seahorse XF Analyzer was used to measure the cellular respiration of ME2-silenced cells, as well as MDSA or EA-treated cells. The OCR was considerably decreased in ME2-silenced HEK293T cells (Fig. 6a), as were the basal and maximum respiration (Figs. 6b, c), the amount of ATP produced by oxidative phosphorylation (Fig. 6d), and the spare respiration capacity (Fig. 6e), demonstrating that ME2 silence substantially reduces cellular respiration and ATP production.

The OCR was reduced in EA-treated cells in a dose-dependent manner (Fig. 6f), as were the baseline and maximum respiration (Fig. 6g, h), ATP synthesis (Fig. 6i), and spare respiration capacity (Fig. 6j), suggesting that EA, by inhibiting ME2, considerably reduces cellular respiration and ATP synthesis. MDSA also had the same kind of effects as EA (Figs. 6k–6o) but was even more effective; for example, reducing ATP synthesis with MDSA requires 25 µM (Fig. 6n), whereas with EA, it required more than 25 µM (Fig. 6i).

Additionally, OCR experiments were conducted to assess the efficacy of EA or MDSA on H1299 and MCF-7 cancerous cell lines (Figs. 5e–n and S13e–n, respectively). It was evident that EA induced OCR changes in H1299 cells (Fig. 5e), as indicated by a decrease in the basal and maximum respiration (Fig. 5f, g, respectively), ATP production (Fig. 5h), and the spare respiration capacity (Fig. 5i). MDSA induced similar OCR alterations in H1299 cells as EA (Fig. 5j), as evidenced by a decrease in the basal and maximum respiration and ATP production (Figs. 5k–m, respectively), but not the spare respiration capacity (Fig. 5n). MCF-7 cells, unlike H1299 cells, were not sensitive to EA or MDSA in response to OCR (Fig. S13e,j, respectively); the basal and maximum respiration, ATP production, and the spare respiration capacity were not changed by treating with EA or MDSA (Fig. S13f–i and S13k–n, respectively); this finding corresponded to the fact that MCF-7 cells were not responsive to ME2-directed metabolism with EA or MDSA (Fig. S13a–d).

### MDSA or EA-mediated ME2 inhibition suppresses cancer cell migration and invasion

**Fig. 7:**
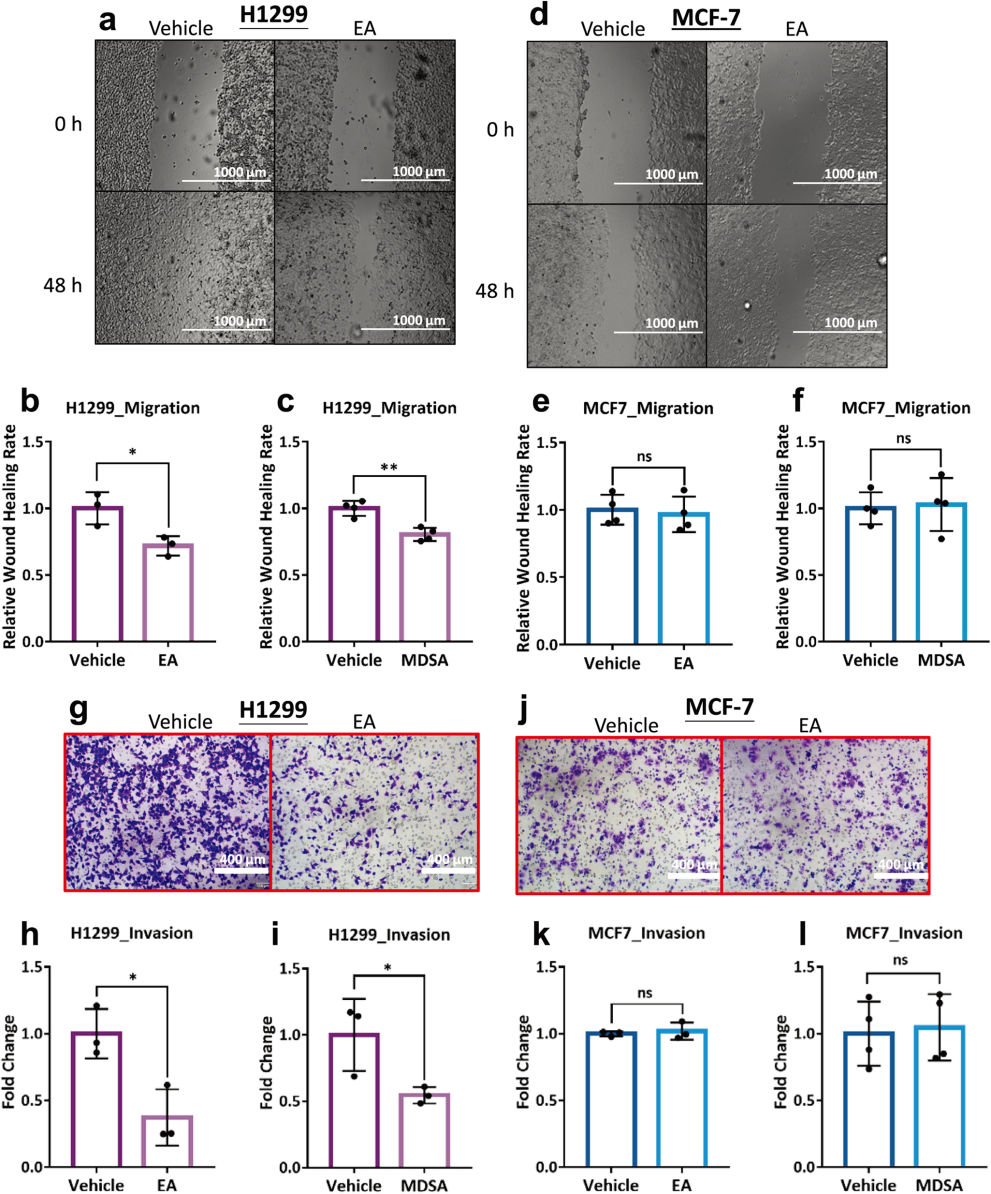
The effect of EA or MDSA on the migration and invasion of H1299 and MCF-7 cells. **a** Cell migration (Wound healing assay) of H1299 cells with EA. **b** Quantitative analysis of relative wound healing rates of H1299 cells in the absence or presence of EA. *N* = 3. Unpaired Student’s *t*-test. ^*^p < 0.05. **c** Quantitative analysis of relative wound healing rates of H1299 cells in the absence or presence of MDSA. *N* = 4. Unpaired Student’s *t*-test. ^**^p < 0.01. **d** Wound healing assay of MCF-7 cells with EA. **e** Quantitative analysis of relative wound healing rates of MCF-7 cells in the absence or presence of EA. *N* = 4. Unpaired Student’s *t*-test. ns, no statistical significance. **f** Quantitative analysis of relative wound healing rates of MCF-7 cells in the absence or presence of MDSA. *N* = 4. Unpaired Student’s *t*-test. ns, no statistical significance. **g** Cell invasion of H1299 cells with EA. **h** Fold changes of invasive H1299 cells in the absence or presence of EA. *N* = 3. Unpaired Student’s *t*-test. ^*^p < 0.05. **i** Fold changes of invasive H1299 cells in the absence or presence of MDSA. *N* = 3. Unpaired Student’s *t*-test. ^*^p < 0.05. **j** Cell invasion of MCF-7 cells with EA. **k** Fold changes of invasive MCF-7 cells in the absence or presence of EA. *N* = 3. Unpaired Student’s t-test. ns, no statistical significance. **l** Fold changes of invasive MCF-7 cells in the absence or presence of MDSA. *N* = 4. Unpaired Student’s t-test. ns, no statistical significance. Error bars are mean ± SD.

We investigated the effects of EA or MDSA on the migration and invasion of H1299 and MCF-7 cancer cells (Fig. 7 and S15). The wound healing assay revealed that EA and MDSA can inhibit the migration of H1299 cells (Fig. 7a and S15a, respectively). The relative wound healing rate of EA- or MDSA-treated H1299 cells was slower than that of untreated cells (Figs. 7b, c, respectively). In contrast, EA and MDSA cannot inhibit MCF-7 cell migration (Figs. 7d and S15b, respectively). Similar wound healing rates were observed between EA- or MDSA-treated MCF-7 cells and untreated cells (Fig. 7e, f, respectively).

The invasion assay demonstrated that EA and MDSA can inhibit cell invasion of H1299 cells (Fig. 7g and S15c, respectively). The fold changes of invasive cells in EA- or MDSA-treated H1299 cells was less than that of untreated cells (Fig. 7h, 7i, respectively). However, neither EA nor MDSA inhibit MCF-7 cell invasion (Fig. 7j and S15d, respectively). The number of invasive cells in MCF-7 cells has not changed considerably after treatment with EA or MDSA (Figs. 7k, l, respectively). In conclusion, EA or MDSA inhibit ME2-associated metabolism, particularly energy metabolism, suggesting that the inhibition of cell migration and invasion by EA or MDSA may be attributable to the suppression of energy production in cancer cells.

## Discussion

ME2 has been crystallized in both open and closed forms with various ligands associated with the active center, allosteric site, and exo site^10,11,42,43^. Our lab previously determined the crystal structures of ME2, revealing that the structure of the ME2_R67Q mutant was an inactivating “dead form” of the enzyme, whereas the structure of ME2_R484W was an overactivating “closed form“^44^. Additionally, ME2 is a dimer of dimers, with each monomer comprising four distinct domains (Domains A, B, C and D). Domain A (residues 23–130) is involved in tetramerization as well as catalysis. An allosteric activator fumarate is bound to the dimer interface of ME2 which contains domain A. Mutations in the fumarate-binding site and domain A frequently result in the inactivation of the enzyme^44^.

**Fig. 8:**
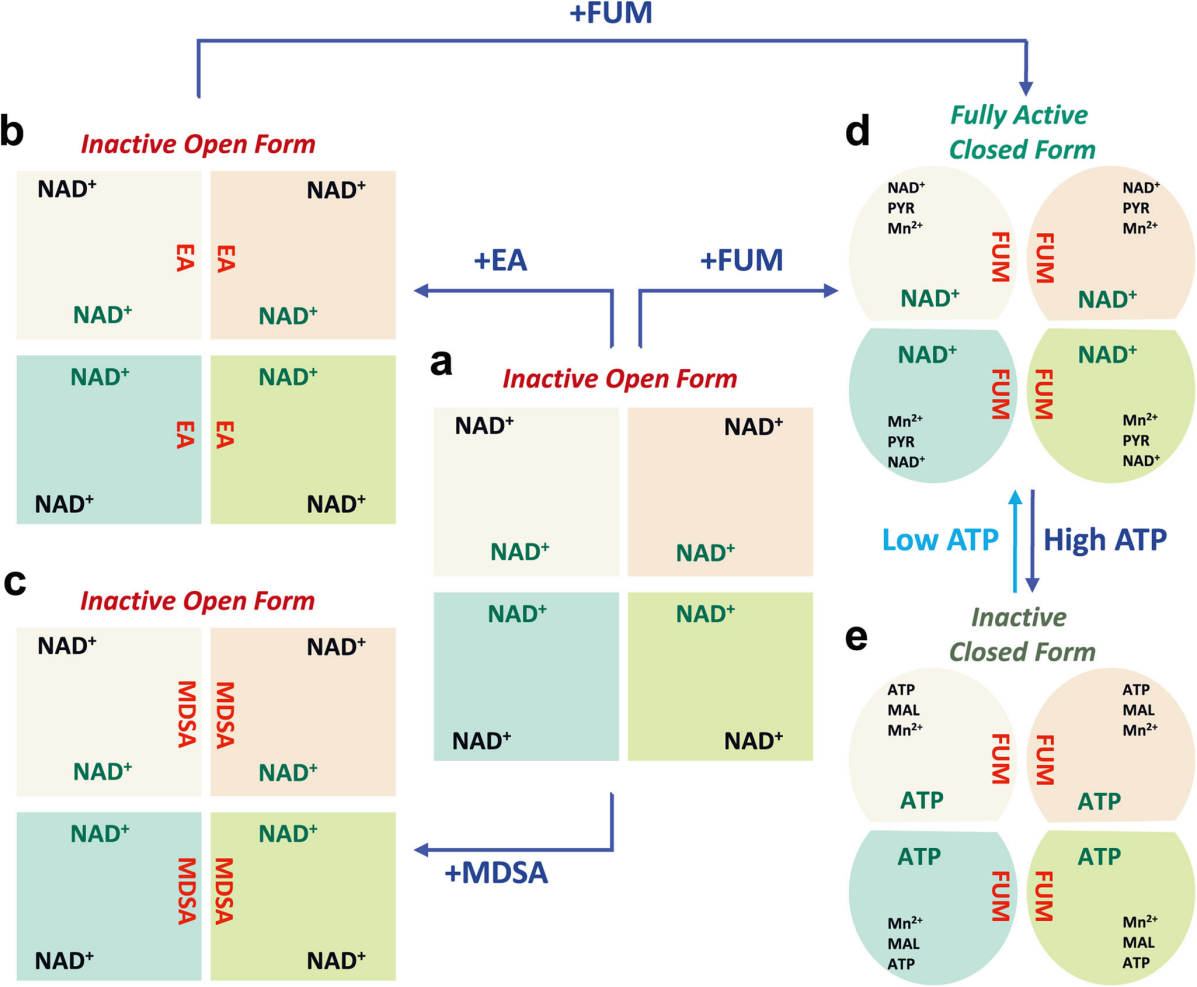
ME2 tetramer undergoes an open-closed transition in the presence of nucleotides, allosteric regulators, fumarate, EA, or MDSA. **a** The ME2-NAD^+^ binary complex presents an inactive open form. **b** The ME2-NAD^+^-EA and **c** ME2-NAD^+^-MDSA ternary complexes also present an inactive open form. EA or MDSA can bind to the allosteric site at the dimer interface, inhibiting ME2 activity by inhibiting the binding of ME2 substrates and thus stabilizing ME2 in an inactive open form. **d** The ME2-NAD^+^-PYR-Mn^2+^-FUM pentary complex (PDB code: 1PJ3) exhibits an active closed form, and **e** The ME2-ATP-MAL-Mn^2+^-FUM pentary complex (PDB code: 1PJ4) displays an inactive closed form. In order to activate ME2 activity, fumarate can bind to the allosteric site at the dimer interface, which promotes the binding of ME2 substrates and thus stabilizes ME2 in a fully active closed form. However, when the cellular ATP level is increased, ATP can compete with NAD^+^ for binding to the active site and exo-site, inhibiting ME2 activity and maintaining ME2 in an inactive closed state. FUM fumarate, PYR pyruvate.

In this paper, we report that three cryo-EM structures of ME2: an open form of ME2 binary complex containing only NAD^+^ (Fig. 8a) and two ME2 ternary complexes containing either EA or MDSA and NAD^+^ (ME2-EA-NAD^+^ or ME2-MDSA-NAD^+^ complexes; Figs. 8b, c, respectively). Based on these cryo-EM structures, we can conclude that EA and MDSA are allosteric inhibitors rather than active-site inhibitors, as they bind to the dimer interface near the fumarate-binding site of ME2 (Fig. 1), and the binding of EA or MDSA prevents ME2 from switching between its open and closed forms, thereby inhibiting its activity. EA and MDSA have unique binding modes that overlap with fumarate’s binding mode at this allosteric site (Fig. 2). Fumarate, an allosteric activator, can largely increase ME2 activity by lowering *K*_0.5,malate_ and *K*_m,NAD,_ thus enhancing the affinity of ME2 for its substrates (Fig. 8d and Table S3). Unlike fumarate binding, which can induce ME2 to transition from open to closed form, however, EA or MDSA binding prevents ME2 from switching from open to closed form (Figs. 8b, c), thereby inhibiting ME2 enzyme activity. Thus, despite the addition of pyruvate, Mg^2+^, NAD^+^ and EA (or MDSA) during cryo-EM sample preparation, we did not obtain an ME2 pentary complex with pyruvate, NAD^+^, Mg^2+^, and EA (or MDSA). This may be because the binding of EA or MDSA locks the ME2 conformation, rendering the active site unsuitable for pyruvate and Mg^2+^ binding. ME2 inhibition by EA or MDSA is reversible, and fumarate can restore ME2 activity that has been inhibited by EA or MDSA by competing for the allosteric site at the dimer interface (Fig. 8b, d)^44^. ME2 activity converts L-malate to pyruvate, which enters the tricarboxylic acid cycle (TCA) and then produces ATP via the respiration chain; thus, when [ATP] is increased, ME2 activity is inhibited (Fig. 8e).

**Fig. 9:**
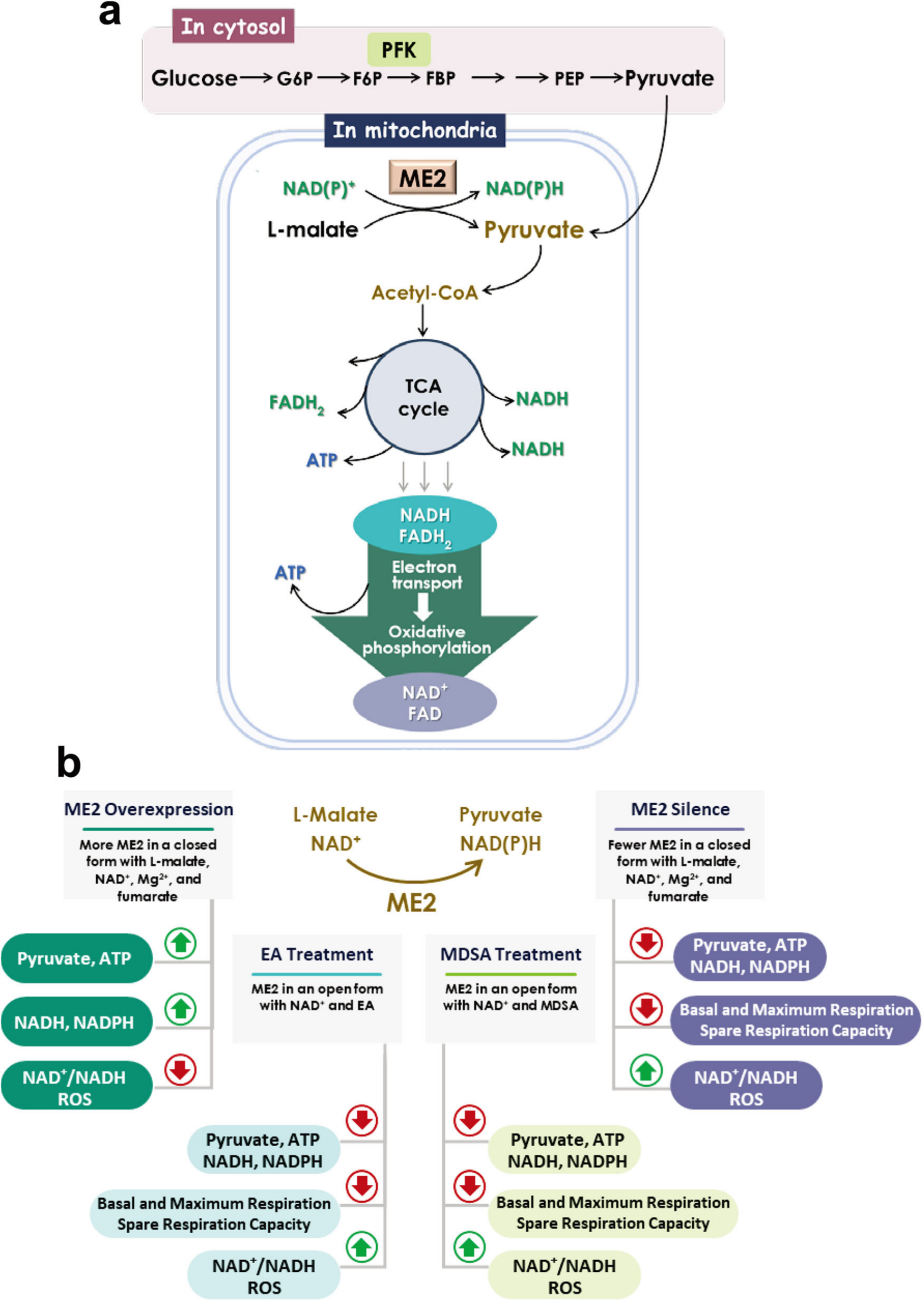
ME2-driven pyruvate and energy metabolism in human mitochondria. **a** A link between the ME2-catalyzed reaction, the tricarboxylic acid cycle (TCA), and oxidative phosphorylation mediated by the electron transport chain. **b** ME2-induced metabolic changes in cells by upregulating or downregulating ME2, and by treating with the ME2 allosteric inhibitors EA and MDSA.

ME2’s regulatory mechanism is quite similar to that of phosphofructokinase (FPK), despite the fact that both enzymes are localized in different places; ME2 is localized in the mitochondria, whereas PFK is localized in the cytosol (Fig. 9a). Of various physiological conditions, ATP is the most effective allosteric inhibitor of ME2 and PFK. Glycolysis generates cytosolic ATP; when ATP levels are high, ATP inhibits PFK, thereby slowing glycolysis. PFK is inactive unless the cell produces fructose-2,6-bisphosphate, its allosteric activator which is bound to PFK and activates it to initiate glycolysis. When [ATP] is depleted, fructose-2,6-bisphosphate is bound to PFK to reactivate the enzyme by lowering the *K*_m_ of PFK’s substrates, followed by the reboot of glycolysis. Similarly, when mitochondrial ATP levels go up, ATP inhibits ME2, thereby decreasing pyruvate production (Fig. 8e). ME2 is also inactive until the cell produces its allosteric activators fumarate, which is bound to ME2 and activates it to generate mitochondrial pyruvate (Fig. 8d). ME2 may be involved in glutaminolysis, and the ME2 reaction produces mitochondrial pyruvate, which is then converted to acetyl-CoA, which is then used to generate additional ATP via the TCA cycle and electron transport chain-mediated oxidative phosphorylation (Fig. 9a). Fumarate is a component of the TCA cycle. When [ATP] is depleted, fumarate binds to ME2 and reactivates it (Figs. 8d, e), reintroducing NAD^+^ into the active site, and ultimately producing pyruvate for ATP synthesis. Thus, both ME2 and PFK are energy-sensing enzymes whose activity is regulated by the cell’s energy state. ME1 is not regulated by ATP, which indicates that it is not an energy-sensing enzyme.

Pyruvate can be obtained from two sources: glucose-derived pyruvate from the cytosol and the ME2-catalyzed conversion of mitochondrial L-malate to pyruvate. L-malate can be produced in the mitochondria through glutaminolysis, transamination, or the malate-aspartate shuttle, among other processes (Fig. 9a). Overexpressing ME2 causes an increase in the levels of pyruvate, NADH, and ATP, as well as a decrease in the NAD^+^/NADH ratio, indicating that ME2 is essential for energy metabolism (Fig. 9b). Increasing ME2 activity in the mitochondria increases the level of NADPH there in order to counteract ROS production, which represents a unique function of ME2 in the mitochondria, whereas PFK in the cytosol lacks antioxidative capability (Fig. 9). ME2 silence, on the other hand, results in a decrease in pyruvate and NADH levels, as well as an increase in the NAD^+^/NADH ratio, when compared to the control group. The reduction of NADPH levels results in an increase in the production of ROS (Fig. 9b). Mechanistically, ME2 silence results in a decrease in ATP production, basal and maximum respiration, as well as spare respiration capacity in the cell, indicating that ME2-catalyzed reactions are crucial for cellular respiration and oxidative phosphorylation there, as demonstrated by the results of this study (Figs. 4–6).

Here, we have reported the discovery of two allosteric ME2 inhibitors, EA and MDSA, that are capable of inhibiting ME2 activity and thus decreasing the production of cellular pyruvate, NADH, and ATP. Treatment with EA or MDSA results in a decrease in cellular respiration and oxidative phosphorylation in the cell (Fig. 9b). EA or MDSA treatment also has the additional effect of decreasing NADPH production, which raises the level of ROS in the cell, which may result in cell death. In other words, the antioxidative capability of ME2 is suppressed by EA and MDSA, leading to a decreased NADPH and an increased ROS. Therefore, ME2 has two functions in mitochondria: the first is producing pyruvate and NADH to generate ATP through oxidative phosphorylation, and the second is generating NADPH to counteract ROS.

ME1, in contrast to ME2, lacks an allosteric site and does not have a dual cofactor specificity; instead, it utilizes only NADP^+^ as a cofactor. Therefore, it is understandable that ME2 was more susceptible to inhibition by EA or MDSA than ME1 in vitro (Fig. S1a). Because it has been discovered that EA or MDSA binds to ME2 at the allosteric site, it is possible that the target site for EA or MDSA in ME1 is located at the active site. This is advantageous in terms of the specificity for ME2’s allosteric inhibition by EA or MDSA. It also implies that the decrease in malate-derived ATP production is due to EA or MDSA-induced ME2 inhibition. Furthermore, although EA and MDSA treatment reduced pyruvate production, this was accomplished primarily through inhibition of ME2 activity, rather than through inhibition of glycolysis, which is the primary pathway for pyruvate production. As evidenced by the fact that the levels of PEP and glucose did not change as a result of the treatment (Fig. S14), the inhibition of ME2 by EA or MDSA does not impair glycolysis.

ME2 has long been suspected of being an oncogene, but no small molecule inhibitor has been found to be effective at inhibiting ME2 activity and causing cell death. We have previously demonstrated that EA can induce cellular senescence in the H1299 cell line, which is a lung adenocarcinoma cell^41^. EA and MDSA are effective at inhibiting ME2 activity at submicromolar concentrations, but cells require micromolar concentrations to prevent ME2-driven pyruvate and energy metabolism. As a result, optimizing the uptake of EA and MDSA into cells is a first priority for future ME2-associated disease research.

## Methods

### Protein expression and purification of ME1 and ME2

The human ME2 protein was expressed in *Escherichia coli* BL21 stain using the PRH281 vector under the control of the trp promoter, which was induced with indol-3-acetic acid (IAA). ME2 was purified using ATP agarose affinity chromatography (Sigma, St Louis, MO, USA). The human ME1 protein was expressed in *Escherichia coli* BL21(DE3) using the pET21b vector under the control of the T7 promoter, which was induced with isopropyl -D-1-thiogalactopyranoside (IPTG). ME1 was purified using Ni-NTA agarose affinity chromatography (Sigma, St Louis, MO, USA). Using a 30 KDa cutoff Amicon®Ultra-15 device, the purified malic enzymes were dialyzed and concentrated against a storage buffer containing 30 mM Tris-HCl (pH 7.4) and 2 mM -mercaptoethanol (Merck Millipore, Billerica, MA, USA). The purity of the protein was determined using SDS-PAGE, and the concentration of the protein was determined using a commercial protein assay buffer (Bio-Rad lab, Inc., Hercules, CA, USA) based on the Bradford method, and the absorbance at 595 nm was detected using a multi-mode microplate reader Biotek® (Agilent, Santa Clara, CA, USA).

### ME2 inhibition study

ME2 inhibition was determined by titrating EA or MDSA from 0 to 40 μM in a reaction buffer containing 50 mM Tris-HCl (7.4), 40 mM L-malate, 2 mM NAD^+^, and 10 mM MgCl_2_; ME1 inhibition was determined in a reaction buffer containing 50 mM Tris-HCl (pH 7.4), 15 mM L-malate, 0.2 mM NADP^+^, and 10 mM MgCl_2_. The inhibition of disalicylic and naphthoic acid derivatives against ME2 was determined by titrating a range of inhibitor concentrations (0 to 500 μM) while maintaining the ME2 substrates at 40 mM L-malate, 2 mM NAD^+^, and 10 mM MgCl_2_. To obtain the IC_50_ value, the inhibition curve can be calculated using the following equation:

1 $${Residual}\,{enzyme}\,{activity}( \% )=\frac{100}{1+\frac{{[A]}^{{Hillslope}}}{{{{{{{{\rm{IC}}}}}}}_{50}}^{{Hillslope}}}}$$

where A denotes the inhibitor concentration. The residual enzyme activity (%) is normalized using the maximum (100%) to minimum (0%) curve. The slope of the curve at its midpoint is called the hillslope. The IC_50_ value indicates the concentration of inhibitor that inhibits 50% of the enzyme’s activity. Prism 8.0 was used to perform all calculations (GraphPad Software, San Diego, CA, USA).

### Site-directed mutagenesis of human ME2

The plasmid pRH281-ME2 was amplified using mutagenic primers (Table S4) for 16–18 thermocycles with a *pfuUltra* high-fidelity DNA polymerase (Agilent, Santa Clara, CA, USA). After digestion with the DpnI restriction enzyme (TaKaRa, Shiga, Japan) to remove the wild-type template plasmid, the DNA products were transformed into *Escherichia coli* XL-10. Finally, autosequencing was used to identify ME2 single mutants.

### Cryo-EM sample preparation

Cryo-EM samples were prepared using a Vitrobot Mark IV (Thermo Fisher Scientific, Waltham, MA, USA) set to 4 °C and 100% humidity. A solution of purified samples (inhibitor-free ME2, ME2-EA, or ME2-MDSA) was applied in an aliquot (3.5 μl) to a glow-discharged Quantifoil R1.2/1.3 holey carbon grid (Quatifoil GmbH, Germany). After a ten-second wait, the grids were blotted with filter paper and immediately immersed in liquid nitrogen-cooled liquid ethane. The grids were blotted for 3.0 s with a blotting force of 0 for the ME2-EA complex (1 mg/ml) and the ME2-MDSA complex (0.5 mg/ml). In the case of ME2 (1 mg/ml) without inhibitor, the grid was blotted for 3.5 s with a blotting force of 5. The cryo-EM grids were then vitrified and stored in liquid nitrogen until imaging.

### Cryo-EM data acquisition

To begin, cryo-EM grids were inspected using a 200 kV Talos Arctica transmission electron microscope equipped with a Falcon III detector (Thermo Fisher Scientific, Waltham, MA, USA). The images were captured in linear mode at a nominal magnification of 92,000×, which corresponds to a pixel size of 1.1 Å/pixel and a defocus setting of −3.0 μm. Suitable cryo-EM grids were stored and recovered in liquid nitrogen until data were collected using the Titan Krios transmission electron microscope (Thermo Fisher Scientific, Waltham, MA, USA). The high-resolution data set was collected automatically on a 300 kV Titan Krios (Thermo Fisher Scientific, Waltham, MA, USA) equipped with an X-FEG electron source using EPU-2.7.0 software. For the ME2-EA and ME2-MDSA complexes, data were collected using a K2 Summit detector in counting mode (gun lens 4, spot size 6, C2 aperture 50 μm) equipped with GIF Bio-Quantum Energy Filters from Gatan. The raw movie stacks were captured at a nominal magnification of 165,000×, which corresponds to a pixel size of 0.82 Å/pixel. The defocus range was set to −1.5 to −2.25 μm, and the Energy Filters’ slit width was set to 20 eV. Sixty frames of non-gain normalized tiff stacks were recorded at a dose rate of ~11 e-/Å2 per second for a total exposure time of 4.5 s, resulting in an accumulated dose of ~50 e^-^/Å^2^ ( ~ 0.83 e^-^/Å^2^ per frame). As with ME2, data were collected using the K3 Summit detector (equipped with GIF Bio-Quantum Energy Filters, Gatan) in super-resolution mode (gun lens 4, spot size 5, C2 aperture 50 μm). The raw movie stacks were captured at a nominal magnification of 81,000×, which corresponds to a pixel size of 1.061 (super-resolution 0.5305 Å/pixel). The defocus range was set to −1.5 to −2.5 μm, and the Energy Filters’ slit width was set to 20 eV. Forty frames of non-gain normalized tiff stacks were recorded at a dose rate of ~14 e^-^/Å^2^ per second for a total exposure time of 2.8 s, resulting in an accumulated dose of ~40 e^-^/Å^2^ ( ~ 1 e^-^/Å^2^ per frame). The parameters used to acquire cryo-EM data are summarized in supplemental Table S2.

### Single-particle image processing and 3D reconstruction

All ME2-EA or ME2-MDSA image stacks acquired in counting mode were imported into Relion for motion correction and dose weighting using MotionCor2^46^ with a 5 × 5 patch without binning (resulting in a pixel size of 0.82 Å /pixel). MotionCor2^46^ was used to motion-correct and dose-weight the super-resolution mode inhibitor-free ME2 image stacks using a 5 × 5 patch and two-fold binning (resulting in a pixel size of 0.83 Å/pixel). The contrast transfer function (CTF) was calculated from the images using CTFFIND4.1 after motion correction and dose weighting^47^. All particles were semi-automatically extracted using cisTEM^48^, and the selected particle coordinates were then imported into Relion 3.0^49^ for particle extraction using a box size of 384 pixels (for ME2-EA and ME2-MDSA complexes) and 256 pixels (for inhibitor-free ME2). Multiple rounds of 2D classification in Relion 3.0^49^ were used to eliminate unsatisfactory 2D class averages, followed by particle selection and extraction. The particles classified in the final round of 2D classification were transferred to cryoSPARC^50^ for generation of ab initio maps. After that, the ab initio maps were imported into Relion^49^ and used as starting references for 3D classification (separated into 3 classes). 3D auto-refinement with D2 symmetry was used to refine the nice 3D classes. After CTF refinement and Bayesian polishing, the polished shiny particles were imported into cryoSPARC^50^ for further 2D classification and 3D heterogeneous refinement without imposing symmetry (C1). After homogeneous and non-uniform refinement with D2 symmetry, particles belonging to the fine 3D classes were refined to a higher resolution. In cryoSPARC, the map was sharpened and the resolution estimated^50^. The overall resolution was determined using the Fourier Shell Correlation (FSC) = 0.143 criterion, while the local resolution was also determined using cryoSPARC^50^. UCSF Chimera was used to visualize 3D density maps^51^. Figure S2 summarizes the details of cryo-EM reconstruction. The procedures for processing single-particle images are summarized in Figs. S3–S5. Table S2 summarizes the statistical data for cryo-EM reconstructions.

### Structure determination and model building

To construct the atomic models for the cryo-EM maps of inhibitor-free ME2 (2.72 Å), ME2-EA (2.72 Å), and ME2-MDSA (2.84 Å), the atomic structure of human ME2 (PDB ID: 1QR6) was rigidly fit into the cryo-EM maps. The conformational differences were manually adjusted in the COOT program^52^ using the “Real Space Refinement Zone” functions in the “Model/Fit/Refine” utility with the “Torsion,” “Plannar Peptide,” “Trans Peptide,” and “Ramachandran” restraints. The PHENIX “Real-space refinement” function was then used to further optimize the atomic model^53^, which included the input atomic model, cryo-EM map, and resolution valve estimated using gold-standard FSC. Following visual inspection in COOT of the PHENIX optimized atomic model, the problematic regions and Ramachandran outliers were manually corrected using the “Real Space Refinement Zone”. Numerous runs of “Real-space refinement” of the atomic model in COOT and PHENIX were performed until no further improvement was obtained. We did not model residues with missing density at the N- and C-termini. The Nicotinamide mononucleotide moiety (NMN) part of the electron density of NAD^+^ is poorly resolved, so we modeled this region mostly base on the geometry restrains. Interesting, part of the MDSA density is relative disappeared in the sharpen map. For MDSA modeling, part of the MDSA density is lost in the sharpened map but can be observed in the unsharpened map (Fig. S16). The electron density of half molecule of MDSA facing out the binding site is less well-defined than the half one in the binding site. We first placed half MDSA in the binding site based on the well-defined density and the other half based on the chemical restraints and traceable density from the unshapen map. It is worth mentioning that the less well-defined density disappeared in the sharpened map suggesting different B-factor within MDSA and the half one facing out the binding site is more flexible. PHENIX’s “Comprehensive validation (cryo-EM)” function was used to validate the atomic model. Table S2 summarizes the validation statistics.

### Cell culture and treatment

Human embryonic kidney 293 cells (HEK293T) were purchased from Thermo Fisher Scientific (Waltham, MA, USA); two human fetal lung fibroblast cells (HFL-1 and MRC-5) and human breast adenocarcinoma cells (MCF-7) were purchased from Bioresource Collection and Research Center (BCRC, Hsinchu, Taiwan). The H1299 human non-small cell lung cancer cell line was acquired from the American Type Culture Collection (ATCC, Manassas, VA, USA). The HEK293T, HFL-1, MRC-5, and MCF-7 cells were cultured in Dulbecco’s Modified Eagle Medium (DMEM) (HyClone^TM^, Cytiva, Marlborough, MA, USA) containing 10% fetal bovine serum (FBS; Sigma, St Louis, MO, USA) and 1% penicillin/streptomycin; the H1299 cell was cultured in RPMI (HyClone^TM^, Cytiva, Marlborough, MA, USA) containing 10% FBS and 1% penicillin/streptomycin. All cell lines were incubated at 37 °C in a humidified incubator containing 5% CO_2_.

### ME2 overexpression by cell transfection

Approximate 70% confluent HEK293T cells (1 × 10^6^ cells) on a 6 cm dish were transfected with the pcDNA3.1-empty (backbone vector) and pcDNA3.1-ME2 by a transfection reagent TransIT-X2^®^ (Mirus Bio LLC, WI, USA) with opti-MEM^TM^ medium (Gibco, Thermo Fisher Scientific, Waltham, MA, USA, Waltham, MA, USA) for 24 h at 37 °C in a humidified incubator containing 5% CO_2_. The pcDNA3.1-empty (Plasmid #52535) was purchased from Addgene (Cambridge, MA, USA) and the pcDNA3.1-ME2 was constructed by inserting the ME2 gene into the vector. Immunoblotting was used to determine the ME2 expression level in the cell.

### Protein knockdown of ME2 by shRNA technology

The lentiviral transfection particle was used to deliver the lentiviral vector pLKO_005 with short hairpin RNA (shRNA). The control vectors pLKO-shCon (TRC2.Void, ASN0000000001) and pLKO-shME2 (TRCN0000294007) were obtained from the National RNAi Core Facility (Academia Sinica, Taipei, Taiwan) and the hairpin sequences of shRNA were as follows: shCon, 5′-CCGGAGTTCAGTTACGATATCATGTCTCGAGACATTCGCGAGTAACTGAACTTTTTT-3′; shME2: 5′- CCGGAGTTCTTACAGAGCTACTAAACTCGAGTTTAGTAGCTCTGTAAGAACTTTTTTG-3′. On 6-well plates, approximately 30% confluent HEK293T (3 × 10^5^ cells/well) cells were treated for 48 h with a lentiviral particle solution at 37 °C in a humidified incubator containing 5% CO_2_. Puromycin (3 μg/ml) was used to eliminate untransfected cells. Using immunoblotting, the ME2 expression level in the cell was determined.

### Measurement of pyruvate and NADPH concentrations in cells

The concentrations of cellular pyruvate and NADPH were determined by the Pyruvate Colorimetric/Fluorometric Assay Kit and PicoProbe™ NADPH Quantitation Fluorometric Assay Kit, respectively (K609-100 and K349-100; BioVision, Milpitas, CA, USA). On 6 cm plates, HEK293T (1.5 × 10^6^ cells), MRC-5 (3 × 10^5^ cells), HFL-1 (3 × 10^5^ cells) and H1299 (3 × 10^5^ cells) and MCF-7 (6 × 10^5^ cells) were cultured for 24 h in medium containing 20 mM L-malate at 37 °C with 5% CO_2_. Cells were treated with EA or MDSA for 48 h. After harvesting, cells were sonicated in 100 µL phosphate-buffered saline (PBS). After centrifugation, the supernatant was deproteinized using a 10 kDa spin column (Acrodisc® syringe filter, Pall Life Sciences) and analyzed using working mixtures in a 96-well plate with the Biotek® multi-mode microplate reader to detect fluorescence at Ex/Em=535/587 nm (Agilent, Santa Clara, CA, USA).

### Measurement of NAD^+^ and NADH concentrations in cells

NAD^+^ and NADH levels were determined using the NAD/NADH-Glo^TM^ Assay (G9071; Promega, Madison, WI, USA). On 6 cm plates, HEK293T (1.5 × 10^6^ cells), HFL-1 (3 × 10^5^ cells), and MRC-5 (3 × 10^5^ cells) were treated for 48 h with 25 µM EA or MDSA at 37 °C with 5% CO_2_. HEK293T (2 × 10^6^ cells in 100 µL PBS), HFL-1 and MRC-5 (2 × 10^5^ cells in 100 µL PBS) were sonicated, and the supernatants were deproteinized using the 10 kDa spin column (Acrodisc® syringe filter, Pall Life Sciences). The supernatants (2 × 10^4^ cells in 100 µL PBS) were mixed with an equal volume of detection reagent in a 96-well plate, and the levels of NAD^+^ and NADH were determined using luminescent signals from the Biotek® multimode microplate reader (Agilent, Santa Clara, CA, USA).

### Measurement of ATP and ROS concentrations in cells

On 6 cm plates, HEK293T (1.5 × 10^6^ cells), HFL-1 (3 × 10^5^ cells) and MRC-5 (3 × 10^5^ cells) were treated with 25 µM EA or MDSA at 37 °C with 5% CO_2_ for 48 h. H1299 (3 × 10^5^ cells) and MCF-7 (6 × 10^5^ cells) were treated with 150 µM EA or MDSA at 37 °C with 5% CO_2_ for 48 h. The ATP content of cells was determined using a CellTiter-Glo® 2.0 Cell Viability Assay (G9242; Promega, Madison, WI, USA). 2 × 10^4^ cells were re-suspended in 100 µL PBS and reacted with an equal volume of the assay buffer. The luminescence was detected using the Biotek® multi-mode microplate reader (Agilent, Santa Clara, CA, USA). The intracellular ROS levels were determined using a ROS Detection Assay Kit (ab287839; Abcam, Cambridge, UK). 1.5 × 10^5^ cells were incubated for 45 min at 37 °C in the dark with a diluted ROS buffer. Following a wash with PBS buffer, the cells were re-suspended in the 150 µL ROS buffer and the fluorescence at Ex/Em=495/529 nm was detected with the Biotek® multi-mode microplate reader (Agilent, Santa Clara, CA, USA).

### Measurement of the oxygen consumption rate (OCR) in mitochondria

The HEK293T cells (3.75 × 10^4^ cells), H1299 (1.75 × 10^4^ cells) and MCF-7 (2.8 × 10^4^ cells) were seeded and incubated in Agilent Seahorse XF24 cell culture microplates for 24 h 37 °C with 5% CO_2_. HEK293T cells were then exposed to 0, 25, and 50 µM EA and MDSA for 4 h, while H1299 and MCF-7 cells were exposed to 0, 75, and 150 µM EA and MDSA for 3 h. After 30 min of incubation at 37 °C in a non-CO_2_ incubator, the growth medium was replaced with a base medium (Agilent, Santa Clara, CA, USA) containing 1 mM pyruvate, 4 mM glutamine, and 1 mg/mL D-glucose. Through a 24 min time interval, the cells were sequentially treated with 5 µM oligomycin A, 2 µM FCCP, and 1 µM ronstone/antinomycin A. The oxygen consumption rate (OCR) was determined using a Seahorse XFe24 Analyzer in conjunction with the Seahorse XF Cell Mito Stress Test (Agilent, Santa Clara, CA, USA). The experimental data were analyzed using the Wave2.6 control program (Agilent, Santa Clara, CA, USA).

### Kinetic assay of malic enzymes

The reaction mixture for ME2 enzyme activity was composed of 10 mM MgCl_2_, 40 mM L-malate, and 2 mM NAD^+^ in 50 mM Tris-HCl (pH 7.4). The *K*_0.5,malate_ and *K*_m,NAD_ values were determined by titrating a range of L-malate and NAD^+^ concentrations, respectively, while maintaining saturated levels of other compounds. A UV/VIS spectrophotometer (Lambda 25, Perkin Elmer, MA, USA) was used to monitor enzyme activity by continuously tracing the increases in NADH, which has a notable absorbance at 340 nm. The Michaelis–Menten equation was used to determine the value of *K*_m,NAD_, and the extinction coefficient of 6.22 mM^−1^ cm^−1^ was used to determine the *k*_cat_ value. The following equation was used to calculate the cooperativity of L-malate:

2 $$v=\frac{{V}_{\max }\times {[{malate}]}^{h}}{{{K}_{0.5,{malate}}}^{h}+{[{malate}]}^{h}}$$

where *v* denotes the initial velocity, *V*_max_ denotes the reaction’s maximum rate, *K*_0.5_ denotes the substrate concentration at half-maximal velocity, and *h* denotes the hill coefficient, which signifies the degree of cooperativity. Prism 8.0 was used to perform all calculations (GraphPad Software, San Diego, CA, USA). Fumarate activation was determined by titrating a range of fumarate concentrations (0 to 6 mM) while maintaining 15 mM L-malate, 1 mM NAD^+^, and 10 mM MgCl_2_.

### Immunoblotting

The cells were lysed with RIPA Lysis buffer (Promega, Waltham, MA, USA). The protein concentration of supernatants was determined using the Bradford method after they were homogenized and centrifuged. The supernatants (50 μg) were separated by SDS-PAGE and transferred to the PVDF blotting membrane. The PVDF was blocked with blocking buffer and incubated for 24 h at 4 °C with customized anti-human ME2 antibodies (0.5 μg/ml) (MDbio Inc., Taipei, Taiwan) and anti-actin antibodies (1 μg/ml) (Arigo Biolaboratories, Hsinchu, Taiwan), followed by an hour at 25 °C with the second antibody labeled with horseradish peroxidase. Finally, the labeled antibodies reacted with enhanced chemiluminescence buffer, and the luminescence was detected using an ImageQuant^TM^ LAS 4000 mini imager (GE Healthcare Life Sciences, Piscataway, NJ).

### Cell viability assay

The relative number of viable cells in a population was determined using the CellTiter-Fluor™ Cell Viability Assay (G6080, Promega, Madison, WI, USA). On 96 well plates, 1 × 10^4^ cells were seeded and incubated for 24 h in 100 µL medium. For an additional 24 h, the cells were treated with various concentrations of EA and MDSA. After replacing 50 µL medium with 50 µL assay reagent in each well, the cells were incubated for 30 min at 37 °C with 5% CO_2_. The viability of the cells was determined using the Biotek® multi-mode microplate reader set to Ex/Em=490/505 nm (Agilent, Santa Clara, CA, USA).

### Circular dichroism spectroscopy

The secondary structure of the ME2 protein was determined using a Jasco J-815 circular dichroism (CD) spectropolarimeter (Jasco Deutschland GmbH, Pfungstadt, Germany). Protein samples (0.3 mg/mL) in 30 mM Tris-acetate (pH 7.4) were detected using a quartz cuvette with a path length of 0.1 cm, and CD spectra data were collected in 0.2 nm increments between 190 and 260 nm. Each CD spectrum was determined using an average of ten individual scans and normalized to the sample concentration.

### Mass analysis

About 70% confluent HEK293T cells (3 × 10^6^ cells) were treated for 48 h with 50 µM EA and MDSA at 37 °C in a humidified incubator containing 5% CO_2_. 4 mL of 80% (vol/vol) methanol was used to extract the cellular metabolites from the cells. After deproteinizing with the 10 kDa spin column (Acrodisc® syringe filter, Pall Life Sciences), the concentration of the extraction solution was normalized against the concentration of DNA or protein. The metabolite extraction solution was evaporated using a nitrogen evaporator to remove the methanol and re-suspended in acetonitrile (ACN) at a concentration of 20% (vol/vol). The liquid chromatographic experiments were conducted on a VanquishTM LC system (Thermo Fisher Scientific, Waltham, MA, USA) equipped with a Cogent Diamond-HydrideTM column (150 × 2.1 mm, 4 µm; MicroSolv Technology Corp., Eatontown, NJ, USA). The mobile phase was composed of (A) 0.01% formic acid and (B) water/ACN 90:10 (vol/vol). The following gradient was used in this study: 0–3 min, 20-20% A; 3–9.5 min, 20–30% A; 9.5–10 min, 30–70% A; 10–11 min, 70-100% A; 11–14 min, 100-100% A; 14-14.1 min, 100–20% A; 14.1–35 min, 20-20% A. The flow rate was 0.2 mL/min. The mass spectrometric analyses were performed on a Thermo Fisher Scientific TSQ AltisTM Triple Quadrupole Mass Spectrometer (Waltham, MA, USA) equipped with an electrospray ionization (ESI) source in positive scan mode using the selected reaction monitoring (SRM) mode. The optimal parameters were as follows: sheath gas flow rate of 35 arbitrary units, auxiliary gas flow rate of 5 arbitrary units, capillary temperature of 325 °C, and spray voltage of 3.5 kV.

### Wound healing assay

H1299 (1.5 × 10^5^ cells) and MCF-7 (3.0 × 10^5^ cells) were seeded into a Costar® 24-well plate (Corning, NY, USA) and cultured for 24 h at 37 °C with 5% CO_2_ to form a confluent cell monolayer. After the cells reached confluence, the medium was removed and PBS buffer was used to wash the cells. The monolayer was then scratched with a 200 µL pipette tip, and the cells were treated with 150 µM EA or MDSA at 37 °C and 5% CO_2_ for 48 h. Using a Lionheart FX automated microscope (BioTek®, Agilent, Santa Clara, CA, USA), the bright field images of the cells were acquired and analyzed.

### Invasion assay

Matrigel® (Corning, NY, USA) was coated in the upper chamber of a 24-well Transwell® plate (Corning, NY, USA) for 24 h (200 µg/ mL matrigel for MCF7, 1000 µg/ mL matrigel for H1299). Matrigel was diluted with the FBS-free medium. Following this, H1299 (1.0 × 10^5^ cells) and MCF7 (1.5 × 10^5^ cells) were seeded in the upper chamber with FBS-free medium and treated for 24 h with 150 µM EA or MDSA. In the interim, the chemoattractant medium containing 10% FBS was added to the lower chamber. An inverted microscope (Olympus Corporation, Tokyo, Japan) was used to capture the cell images, which were then analyzed using Image J^54^.

### Statistics and reproducibility

The data presented denote means±standard deviations (mean±SD). In these instances, the number of biologically independent samples is three or more. The statistical analysis was conducted using the unpaired two-tailed Student’s *t*-test or the one-way analysis of variance (ANOVA) with Dunnett’s test at significance levels of ^*^p < 0.05, ^**^p < 0.01, and ^***^p < 0.001.

### Reporting summary

Further information on research design is available in the Nature Portfolio Reporting Summary linked to this article.

## Supplementary information

Supplementary Information

Description of Additional Supplementary Files

Supplementary Data 1

Reporting Summary

## Data Availability

The cryo-EM structures determined here are deposited in the Electron Microscopy Data Bank (EMDB) under accession codes EMD-33145 (open form of ME2), EMD-33146 (ME2-EA complex), and EMD-33147 (ME2-MDSA complex). The associated molecular models are deposited in the PDB under accession code 7XDE (open form of ME2), 7XDF (ME2-EA complex), and 7XDG (ME2-MDSA complex). All other data generated or analyzed during this study are included in this published article, the Supplementary Data, and the Supplementary Information files. The data sets generated and analyzed during this study are available from the corresponding author upon request.
