# Supplementary material for: Suppression of the human malic enzyme 2 modifies energy metabolism and inhibits cellular respiration
Source: Commun Biol. 2023 May 22;6:548. doi: 10.1038/s42003-023-04930-y (PMC10202922; doi:10.1038/s42003-023-04930-y)
Supplement: Supplementary file 1 — Supplementary Information [file 42003_2023_4930_MOESM1_ESM.pdf]

Supplementary Information for

Suppression of the human malic enzyme 2 modifies energy metabolism and inhibits cellular respiration

Ju-Yi Hsieh, Kun-Chi Chen, Chun-Hsiung Wang, Guang-Yaw Liu, Jie-An Ye, Yu-Tung Chou, Yi-Chun Lin, Cheng-Jhe Lyu, Rui-Ying Chang, Yi-Liang Liu, Yen-Hsien Li, Mau-Rong Lee, Meng-Chiao Ho, and Hui-Chih Hung

Correspondence: Meng-Chiao Ho, and Hui-Chih Hung

E-mail: joeho@gate.sinica.edu.tw; hchung@dragon.nchu.edu.tw

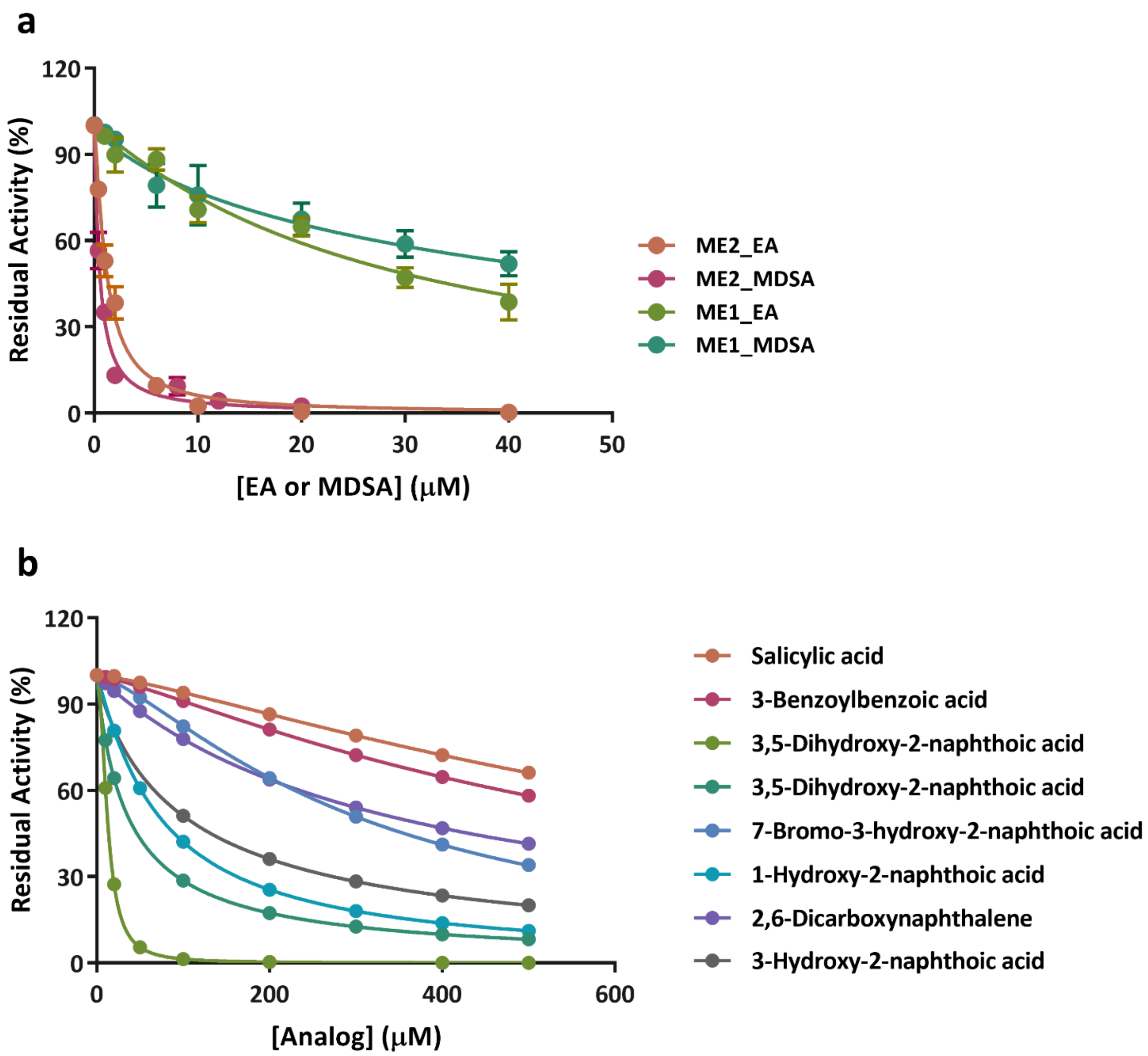

Figure S1. ME2 inhibition plots in the presence of allosteric inhibitors. **a** ME1 and ME2 residual enzyme activity was determined using a range of EA and MDSA concentrations, and all experiments were performed in triplicate or (EA: N = 3, MDSA: N=4; mean ± SD). The IC_{50} values of ME1 and ME2, for EA and MDSA, are indicated. **b** ME2 residual enzyme activity was determined using a series of disalicylic acid and naphthoic acid derivatives in quantities ranging from 0 to 500 μM. All experiments were performed in triplicate. The IC_{50} values are shown in Table S1.

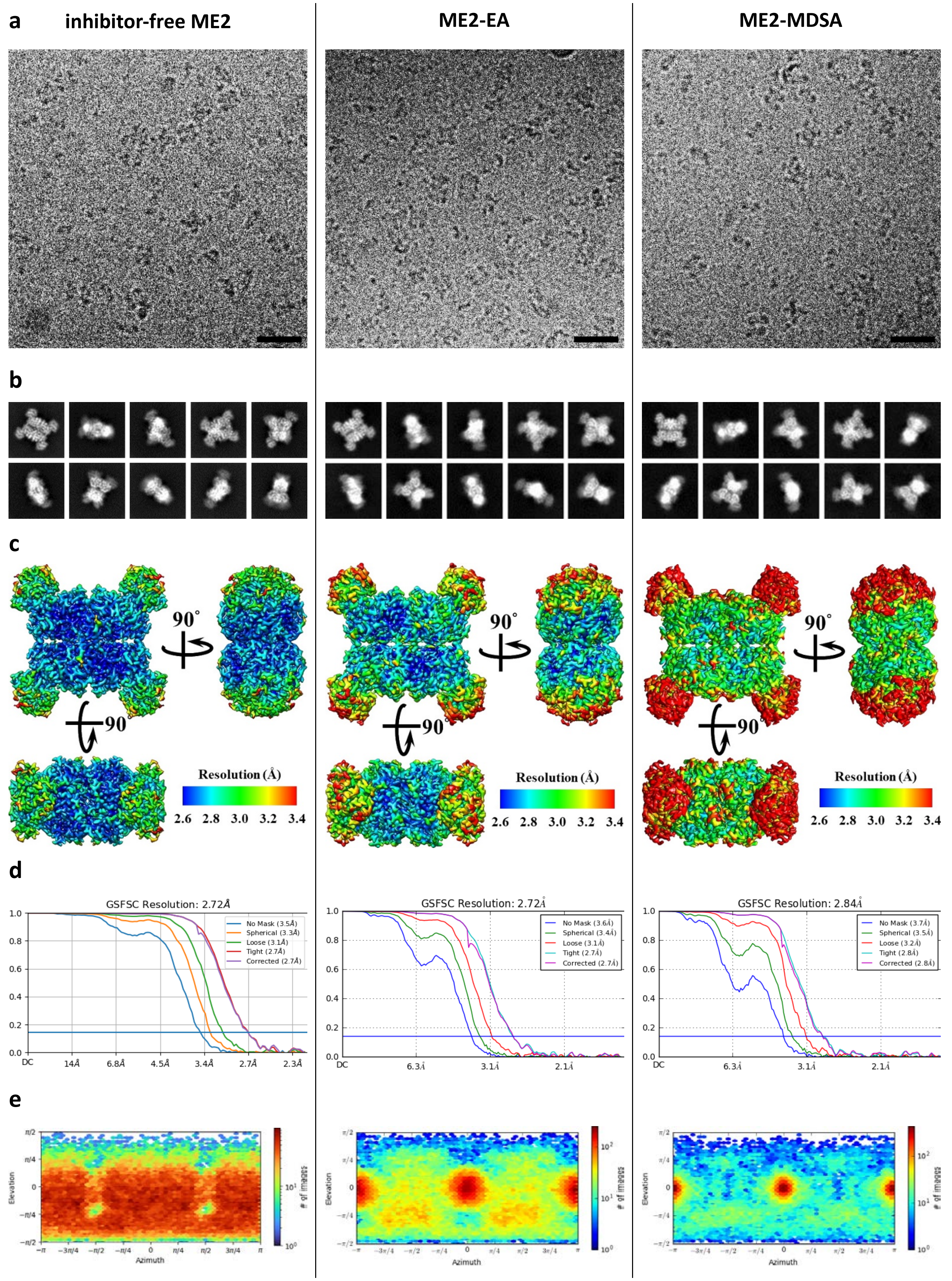

Figure S2. Single-particle cryo-EM analysis of inhibitor-free ME2, EA-ME2, and MDSA-ME2. a Representative cryo-EM image. **b** Reference-free 2D class averages. **c** Resolution maps for the final reconstructions. The maps of inhibitor-free ME2, ME2-EA and ME2-MDSA are contoured at 5.5 σ, 4.5 σ,

and 5.7 σ above the mean, respectively. **d** Gold standard FSC plots for the 3D reconstructions. **e** Euler angle distribution of the particle images.

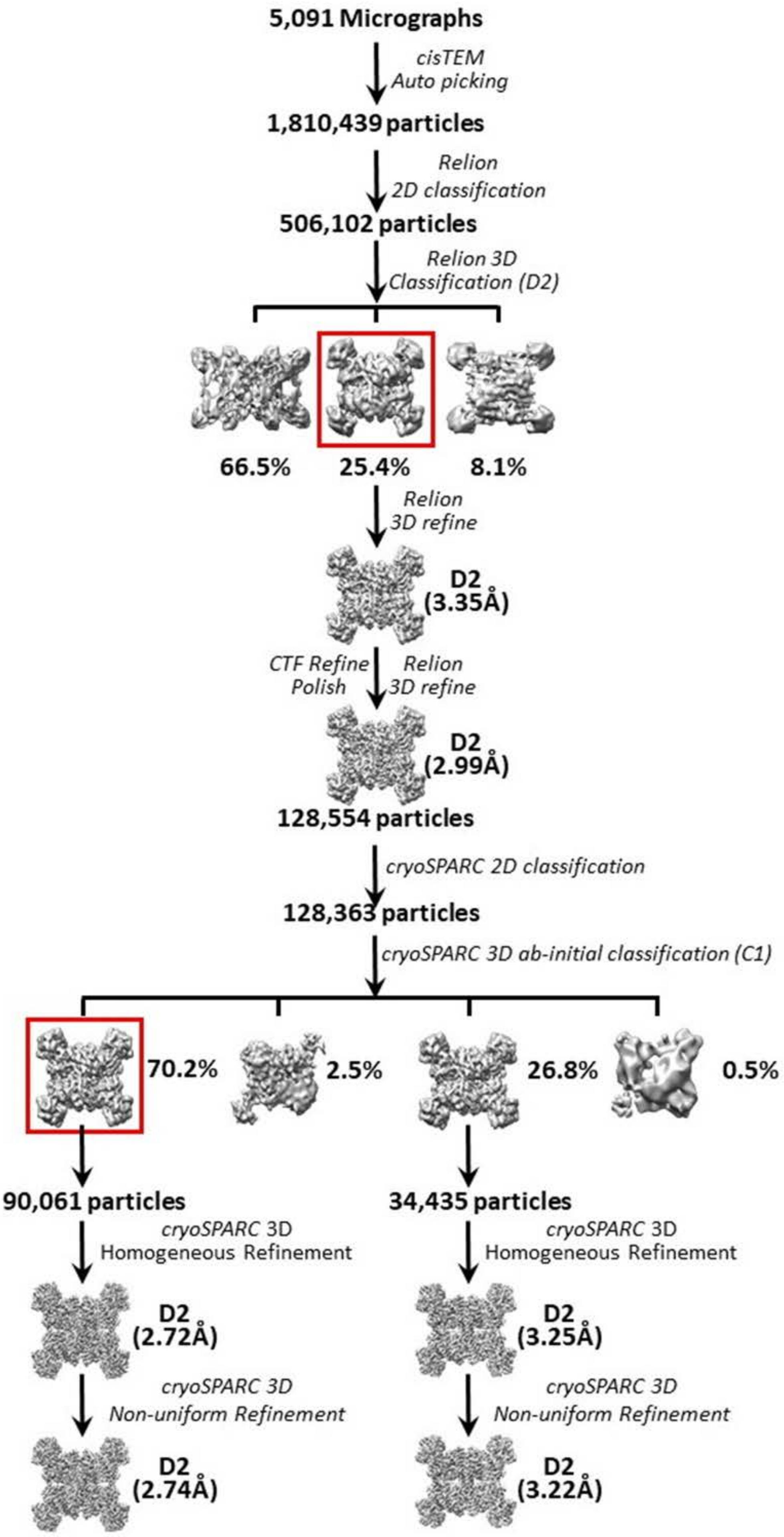

Figure S3. Workflow of data processing of the inhibitor-free ME2.

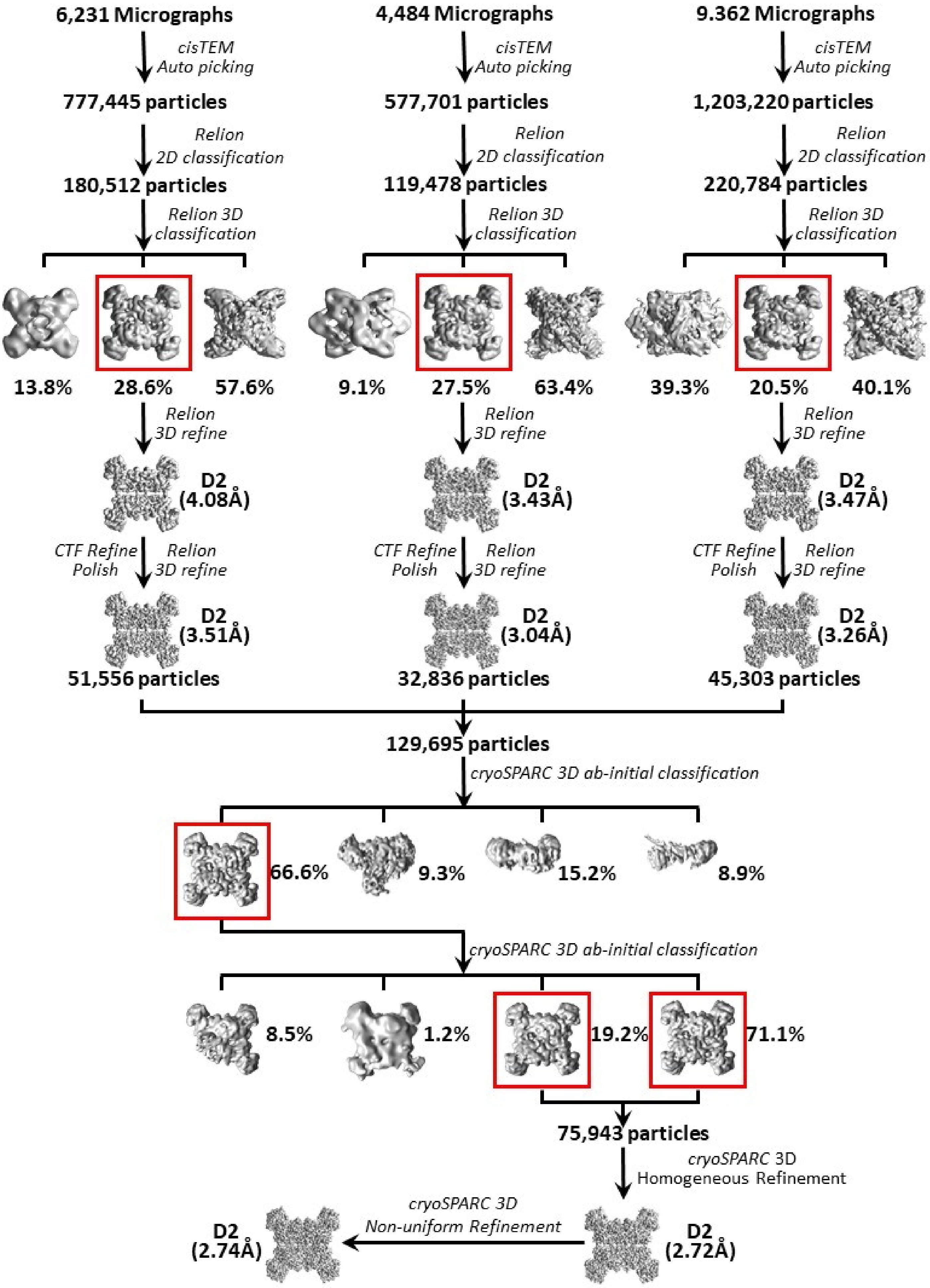

Figure S4. Workflow of data processing of the ME2-EA complex.

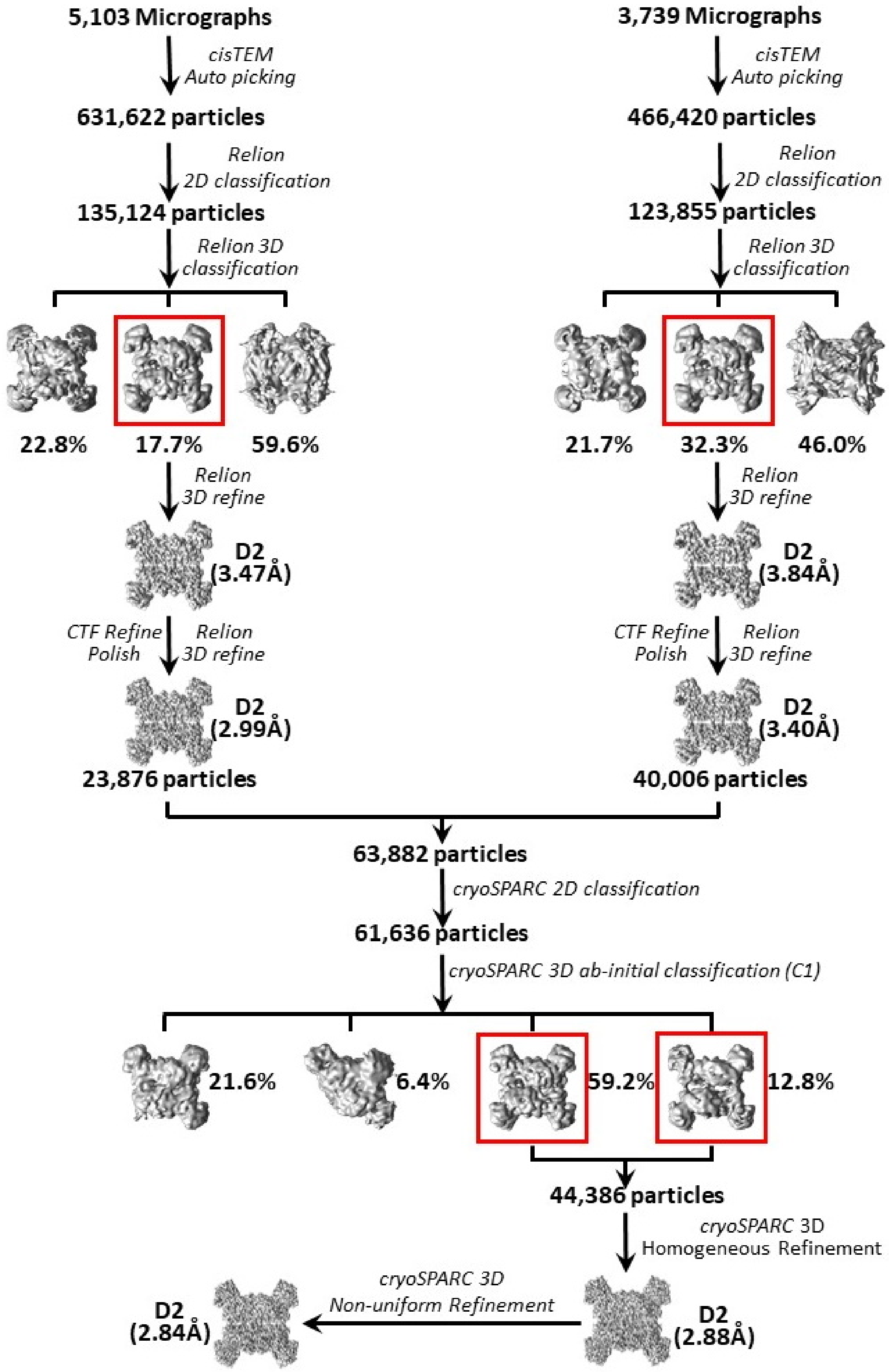

Figure S5. Workflow of data processing of the ME2-MDSA complex.

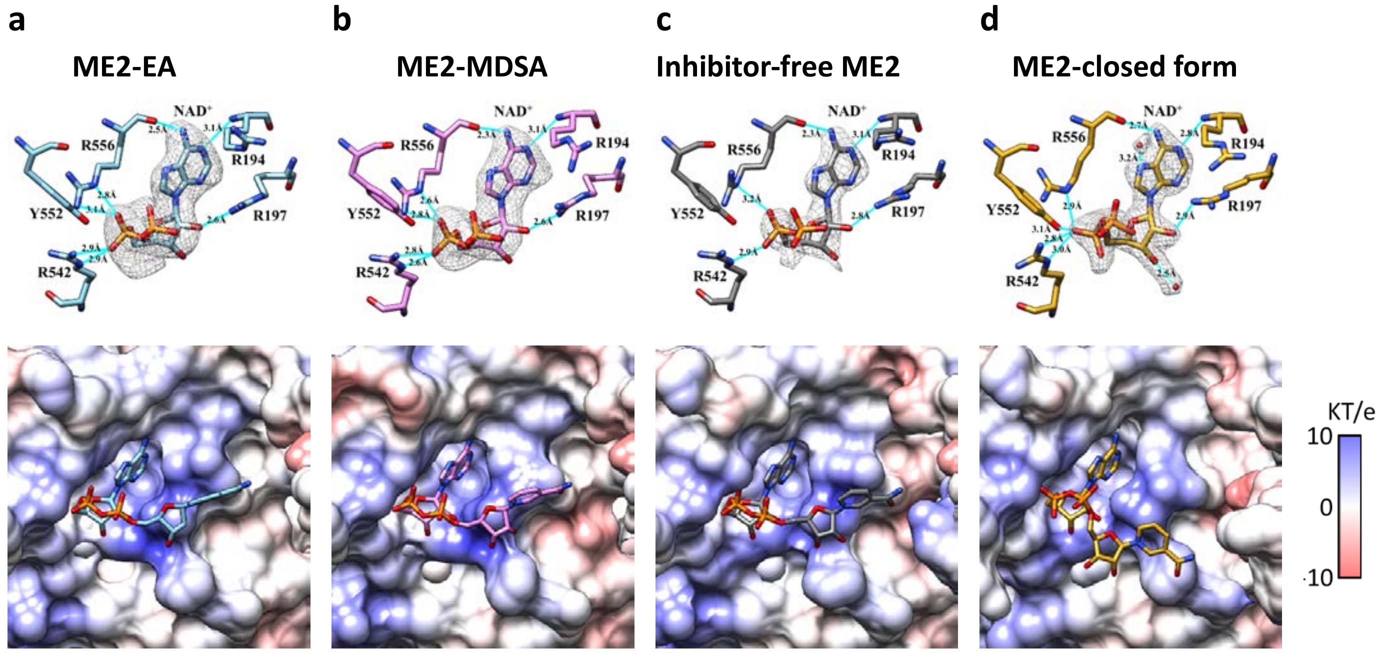

Figure S6. Exo-site NAD^+ coordination of ME2_EA, ME2_MDSA, and inhibitor-free ME2, and closed forms of ME2. The structures illustrate the ligand interactions of exo-site NAD^+ (upper panels) and the Coulombic surfaces surrounding the exo site (lower panels). The gray meshes represent the densities of ligands, and the sticks represent the interacting residues. Hydrogen bonding interactions are indicated by cyan lines, cation-π interactions by green lines, and ion pairs by magenta lines. In upper panels, only the ADP portion of the NAD^+ molecular is shown. **a** The exo-site NAD^+ and the interacting residues are depicted as light blue sticks in the ME2-EA complex. The map is contoured at 6.5 σ above the mean. **b** The exo-site NAD^+ and the interacting residues are depicted as pink sticks in the ME2-MDSA complex. The map is contoured at 7.5 σ. **c** The exo-site NAD^+ and the interacting residues are depicted as gray sticks in the ME2 open form. The map is contoured at 6.5 σ. **d** The exo-site and the interacting residues are depicted as yellow sticks in the ME2 closed form (PDB ID: 1PJ3). The map is contoured at 1.5 σ. Coulombic surfaces were calculated using the default settings in UCSF Chimera[1].

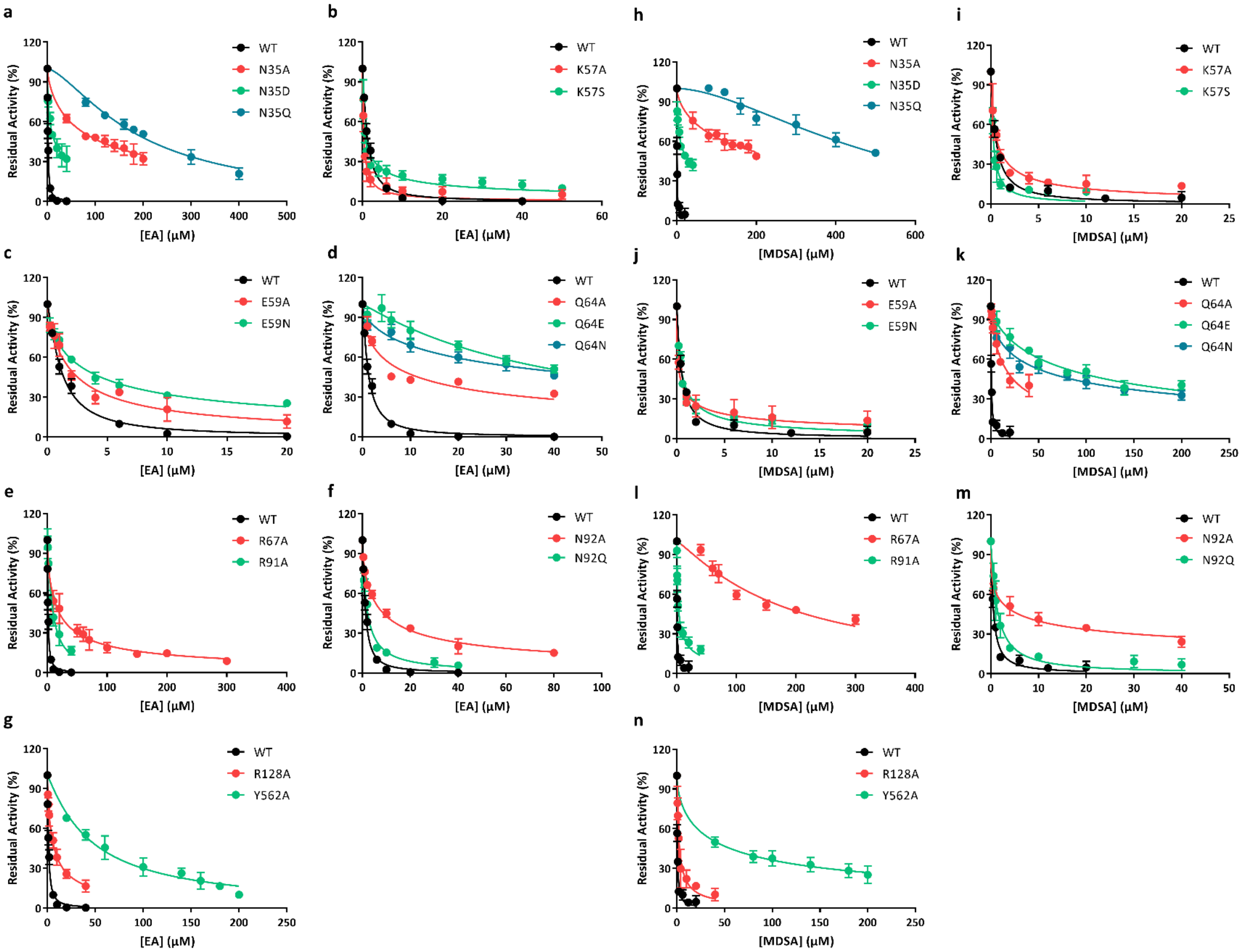

Figure S7. Inhibition plots of ME2 allosteric EA- or MDSA-binding-site mutants. The residual enzyme activity of ME2 allosteric EA- and MDSA-binding-site mutants was determined using a range of EA and MDSA concentrations. **a-g** ME2 inhibition by EA. **h-n** ME2 inhibition by MDSA. **a** and **h** N35 series mutants of ME2. **b** and **i** K57 series mutants of ME2. **c** and **j** E59 series mutants of ME2. **d** and **k** Q64 series mutants of ME2. **e** and **l** ME2-R67A and ME2-R91A. **f** and **m** N92 series mutants of ME2. **g** and **n** ME2-R128A and ME2-Y562A. Figures S7a-S7f, N = 3, mean ± SD. Figures S7g-S7n, N = 3-4, mean ± SD.

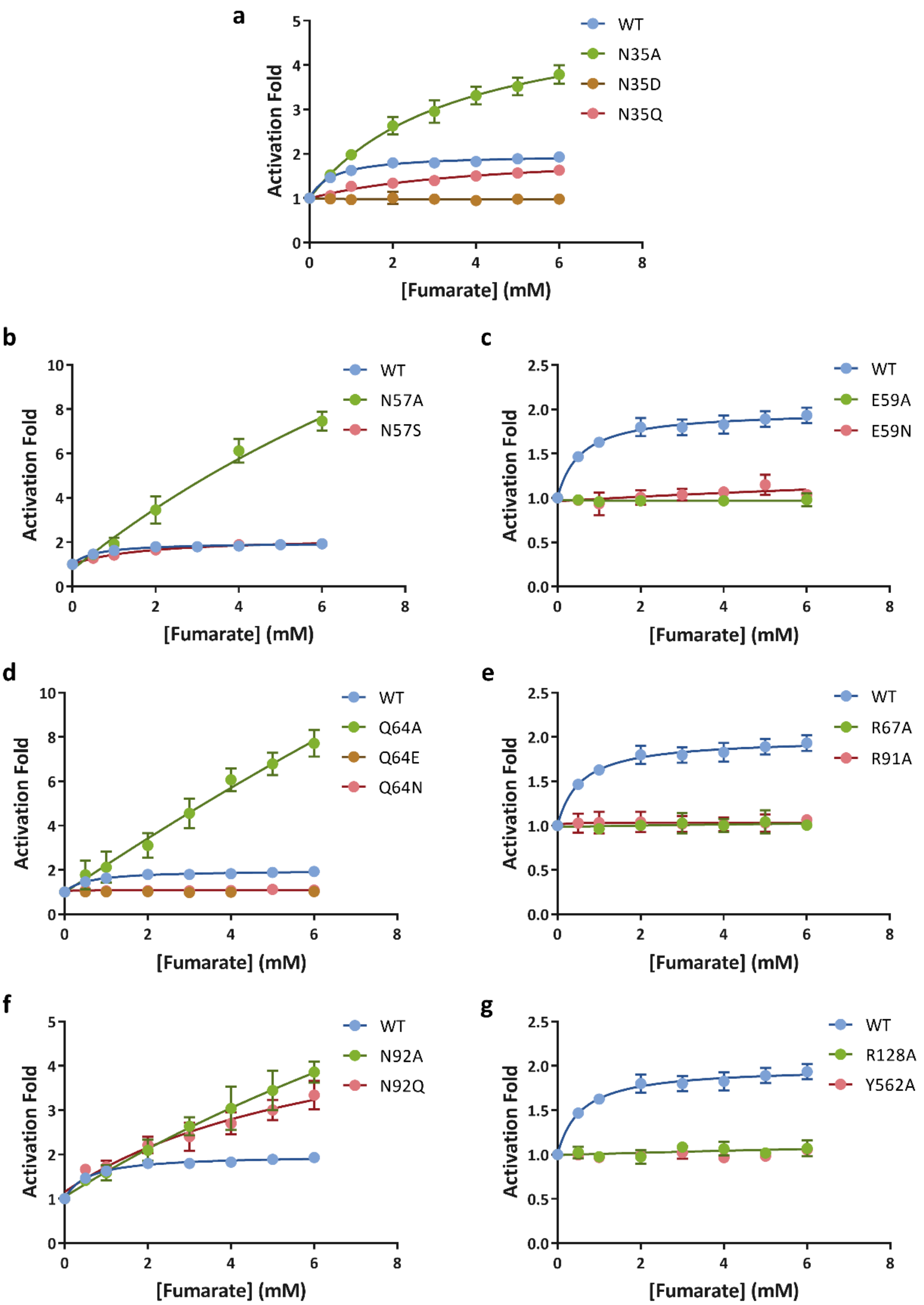

Figure S8. Fumarate activation of ME2 allosteric EA- or MDSA-binding-site mutants. The activation of ME2 allosteric EA- and MDSA-binding-site mutants was evaluated using a range of fumarate concentrations. **a** N35 series mutants of ME2. **b** K57 series mutants of ME2. **c** E59 series mutants of ME2. **d** Q64 series mutants of ME2. **e** ME2-R67A and ME2-R91A. **f** N92 series mutants of ME2. **g** ME2-R128A and ME2-Y562A. Figures S7a-S7c, N = 3, mean ± SD. Figures S7d-S7g, N = 3-4, mean ± SD.

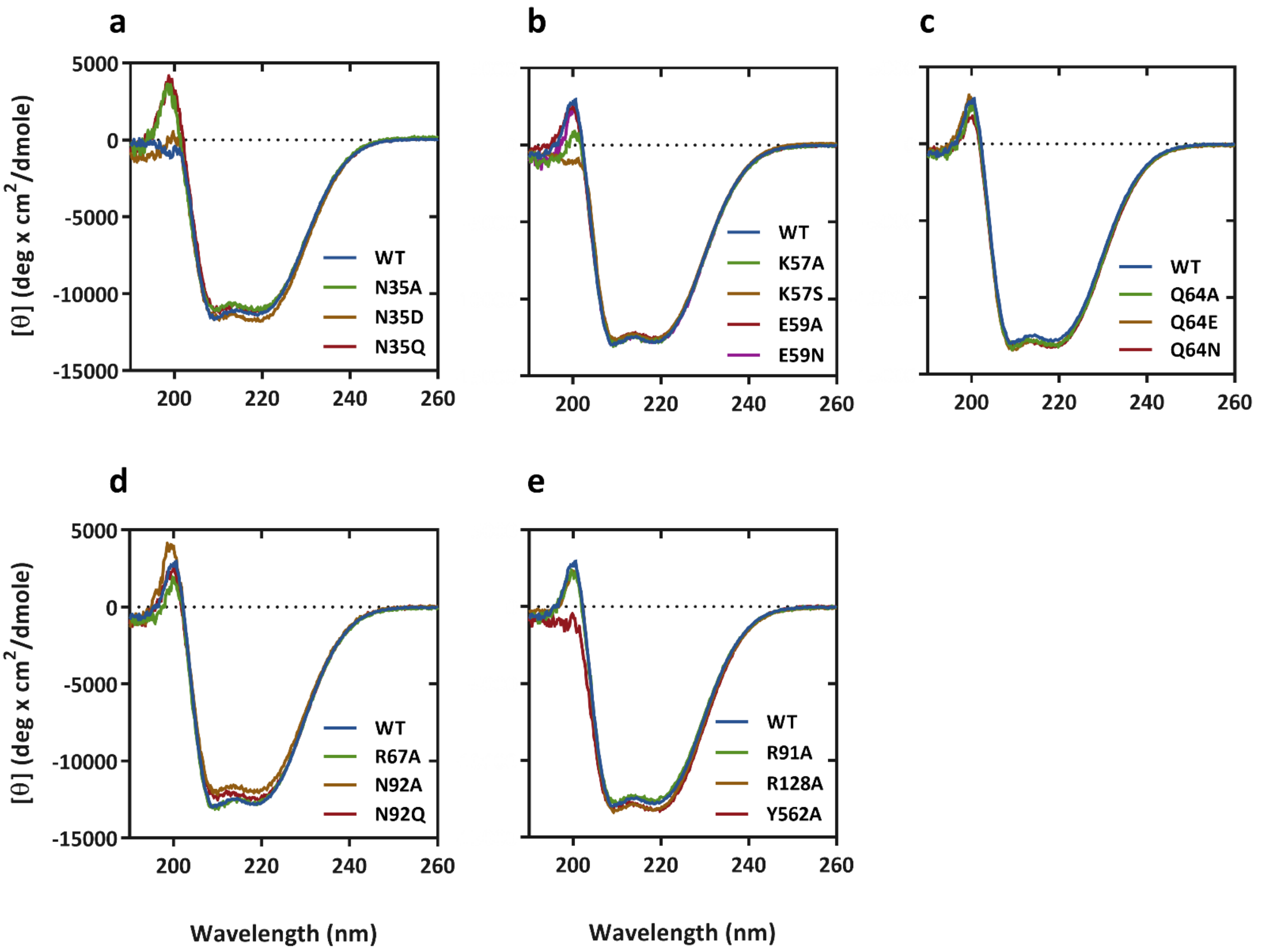

Figure S9. CD spectra of ME2 allosteric EA- or MDSA-binding-site mutants. Circular dichroism (CD) spectropolarimetry was used to determine the secondary structures of ME2 allosteric EA- and MDSA-binding-site mutants. The CD spectrum between 190 and 260 nm can be used to deduce changes in the backbone conformation between WT and mutants. **a** N35 series mutants of ME2. **b** K57 and E59 series mutants of ME2. **c** Q64 series mutants of ME2. **d** ME2-R67A and N92 series mutants of ME2. **e** ME2-R91A, ME2-R128A and ME2-Y562A.

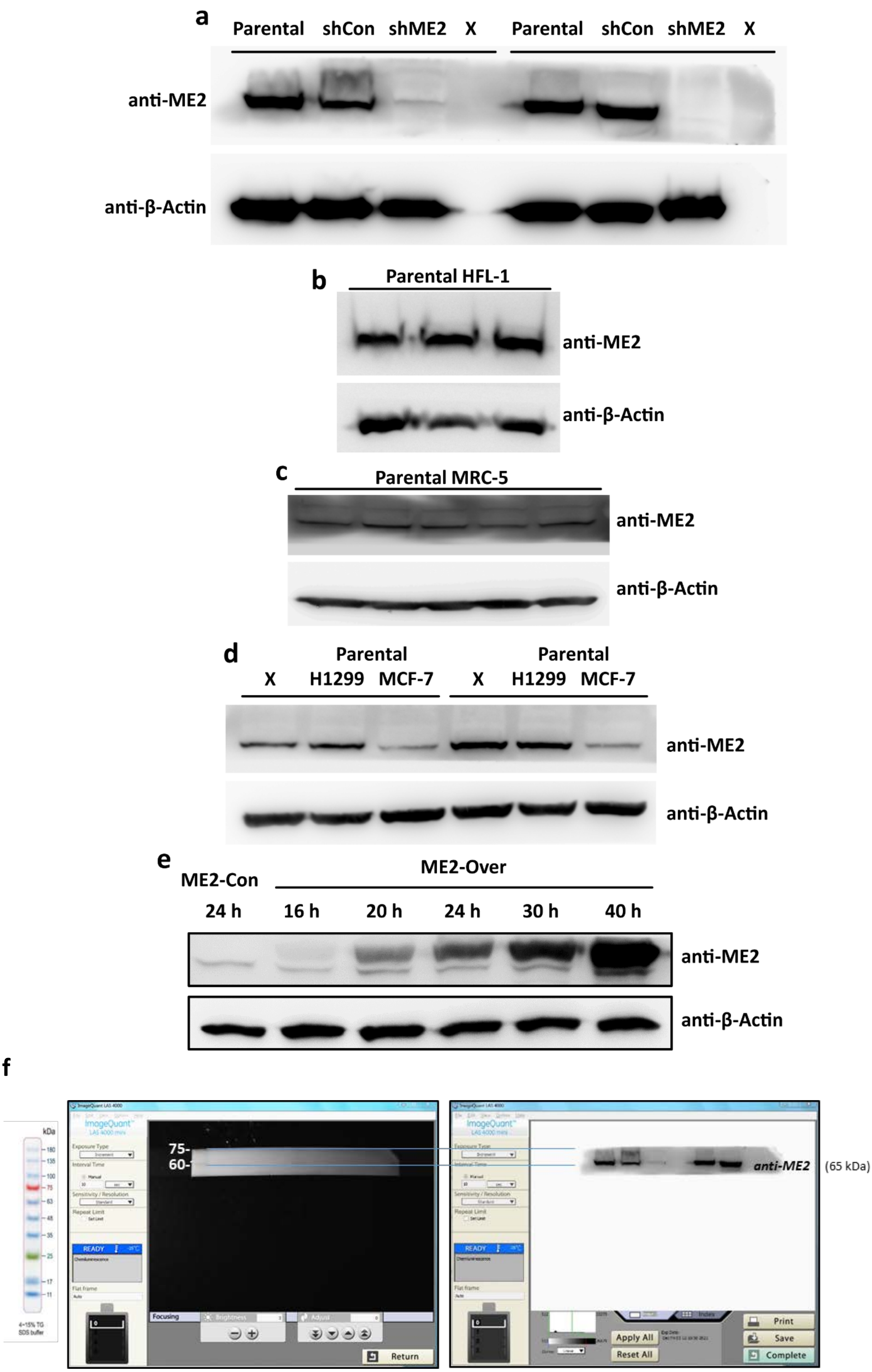
a
Parental shCon shME2 X Parental shCon shME2 X
anti-ME2
anti-β-Actin
b
Parental HFL-1
anti-ME2
anti-β-Actin
c
Parental MRC-5
anti-ME2
anti-β-Actin
d
Parental
X H1299 MCF-7
Parental
X H1299 MCF-7
anti-ME2
anti-β-Actin
e
ME2-Con
ME2-Over
24 h 16 h 20 h 24 h 30 h 40 h
anti-ME2
anti-β-Actin
f
kDa
75-
60-
Focusing
Return
Apply All
Reset All
Print
Save
Complete
anti-ME2
(65 kDa)

g

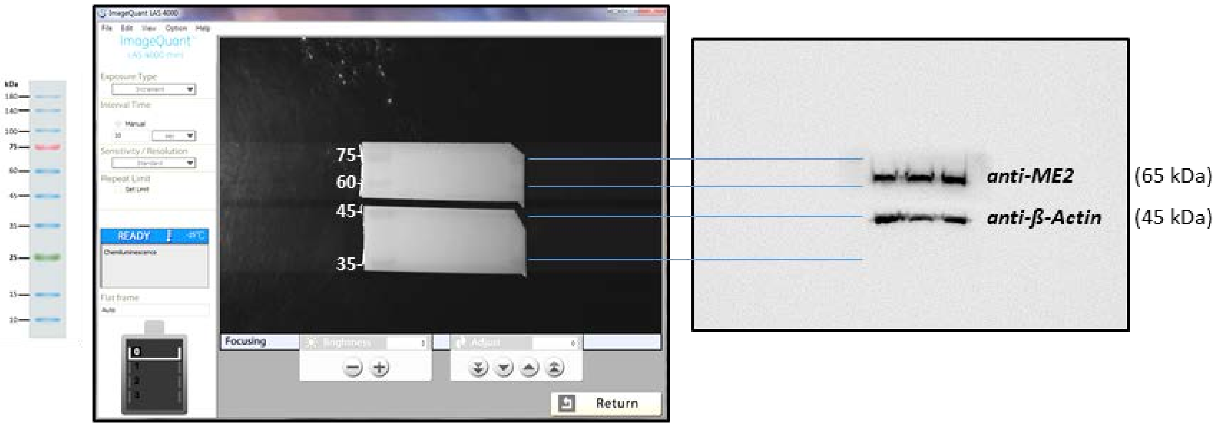
75
60
45
35
anti-ME2
(65 kDa)
anti-ß-Actin
(45 kDa)
Focusing
Return

h

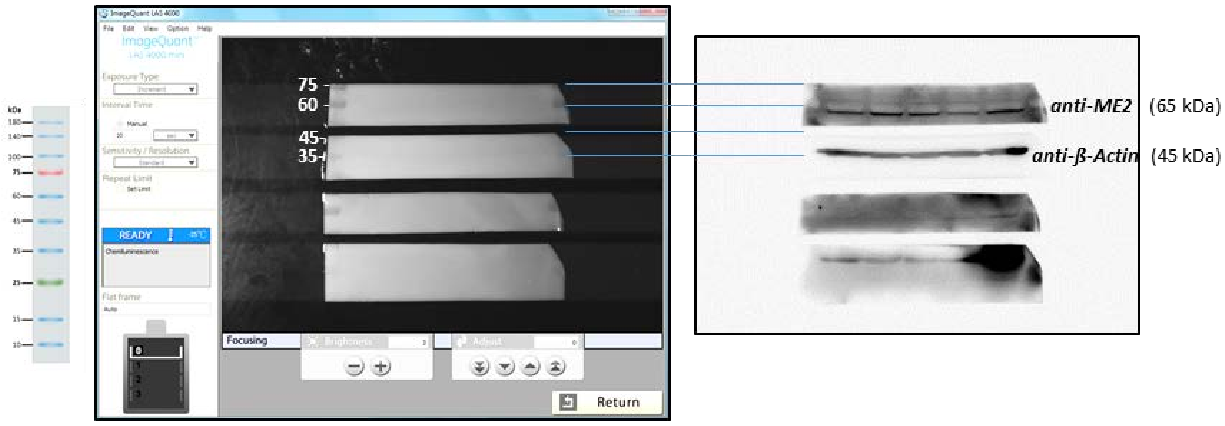
75
60
45
35
anti-ME2
(65 kDa)
anti-ß-Actin
(45 kDa)
Focusing
Return

i

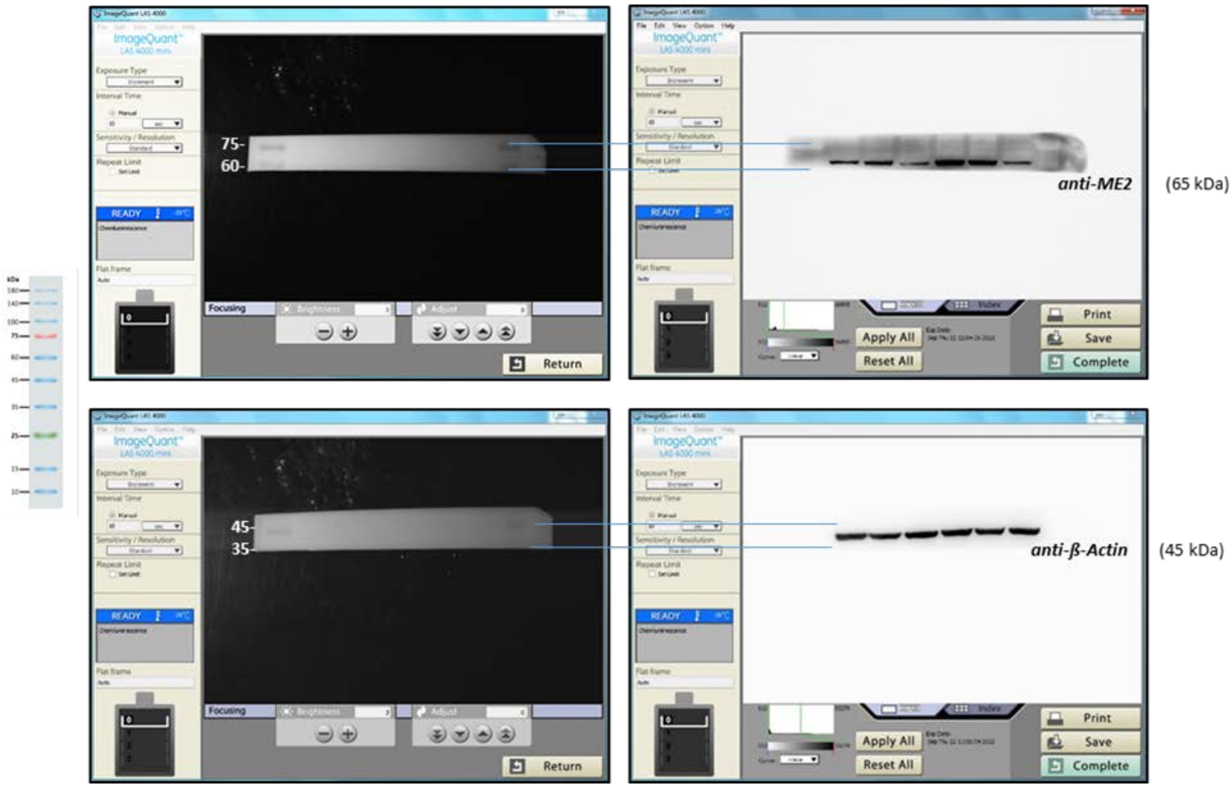
75
60
anti-ME2
(65 kDa)
45
35
anti-ß-Actin
(45 kDa)
Focusing
Return
Print
Save
Complete
Apply All
Reset All

j

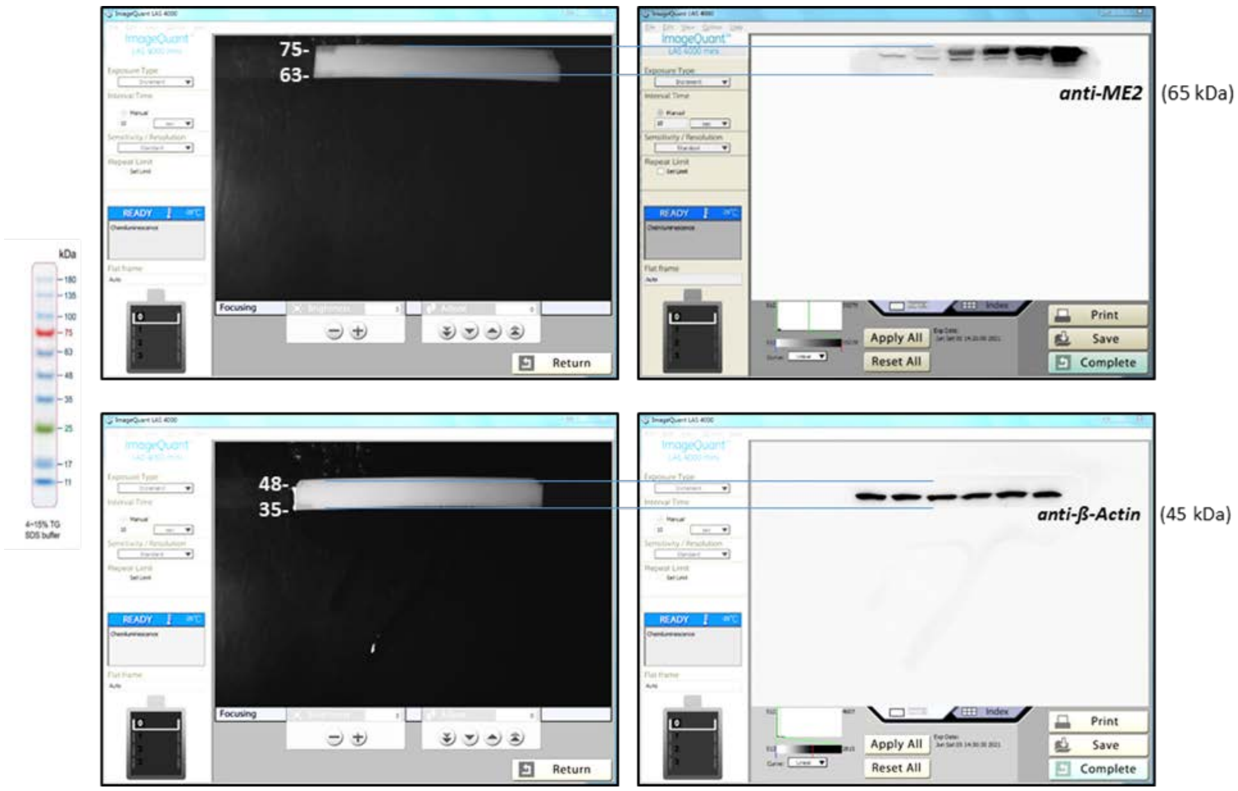

Figure S10. Immunoblots of ME2 in parental, ME2-overexpressing, and ME2-silenced cells. a ME2 protein expression in parental and ME2-silenced HEK293T cell; shCon, cells transfected with the control vector pLKO-shCon; shME2, cells transfected with the plasmid pLKO-shME2. **b**, **c**, and **d** ME2 protein expression in parental HFL-1, MRC-5, H1299, and MCF-7 cells, respectively. **e** ME2 protein expression in ME2-overexpressing cells. ME2-Con, cells transfected with the control vector pcDNA3.1; ME2-Over, cells transfected with the plasmid pcDNA-ME2 and harvested at various time points (0, 16, 24, 30, and 40 hours). ME2 in the cell was detected with immunoblotting against human anti-ME2 antibodies. **f-j** Western blotting of ME2 with protein markers, corresponding to figures S10a-S10e.

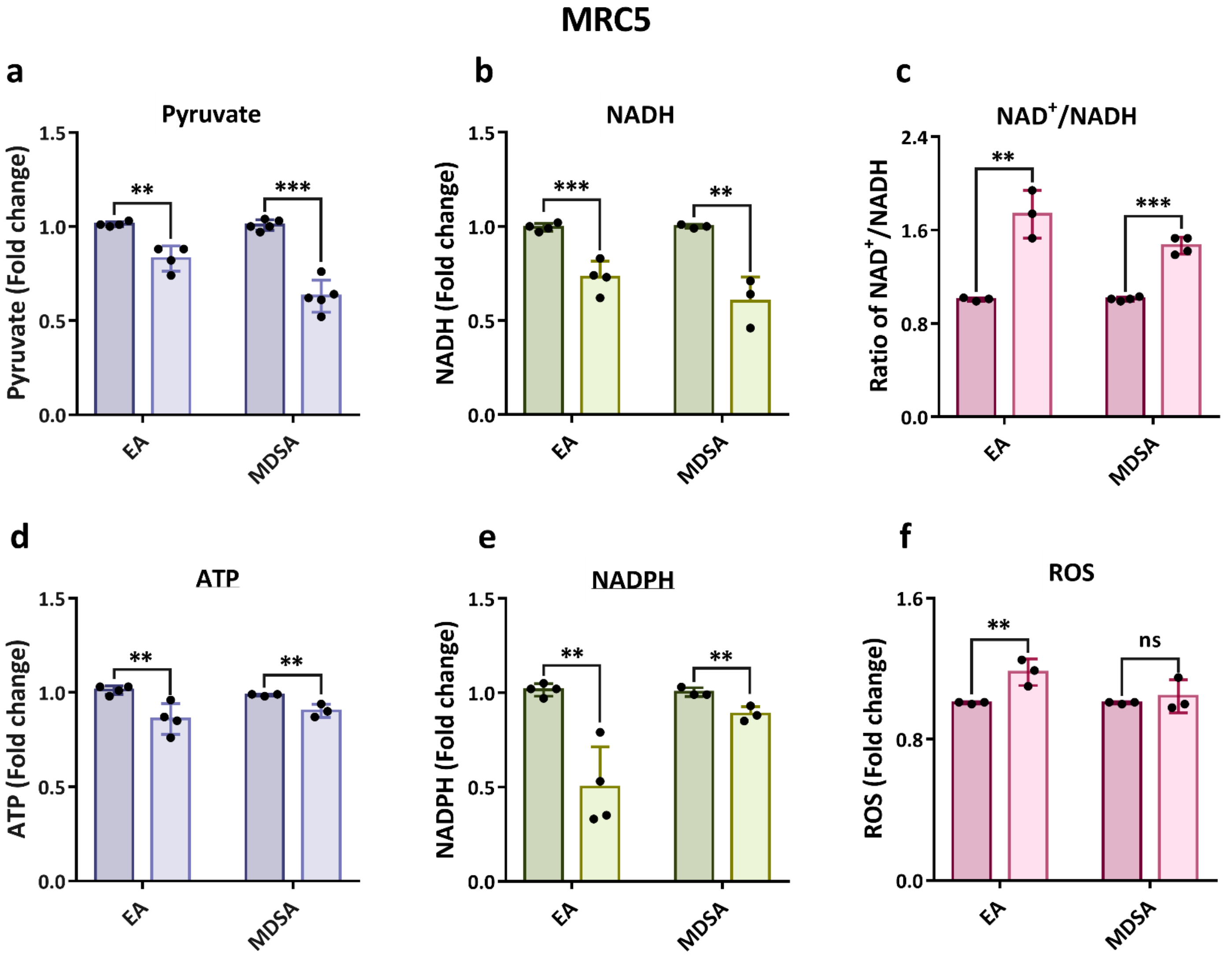

Figure S11. Change in the levels of pyruvate and NADH, the ratio of NAD^+/NADH, ATP, NADPH, and reactive oxygen species (ROS) in MRC-5 cells treated with EA or MDSA. **a** The fold change in pyruvate levels. N = 4-5. Unpaired Student's *t*-test. $^{**}p < 0.01$, $^{***}p < 0.001$. **b** The fold change in NADH levels. N = 3-4. Unpaired Student's *t*-test. $^{**}p < 0.01$, $^{***}p < 0.001$. **c** The fold change in the ratio of NAD^+/NADH. N = 3-4. Unpaired Student's *t*-test. $^{**}p < 0.01$, $^{***}p < 0.001$. **d** The fold change in ATP levels. N = 3-4. Unpaired Student's *t*-test. $^{**}p < 0.01$. **e** The fold change in NADPH levels. N = 3-4, Unpaired Student's *t*-test. $^{**}p < 0.01$. **f** The fold change in ROS levels. N = 3. Unpaired Student's *t*-test. $^{**}p < 0.01$. ns, no statistical significance. The bar graphs illustrate the fold change in the levels of these metabolites after 48 hours. Error bars are mean ± SD.

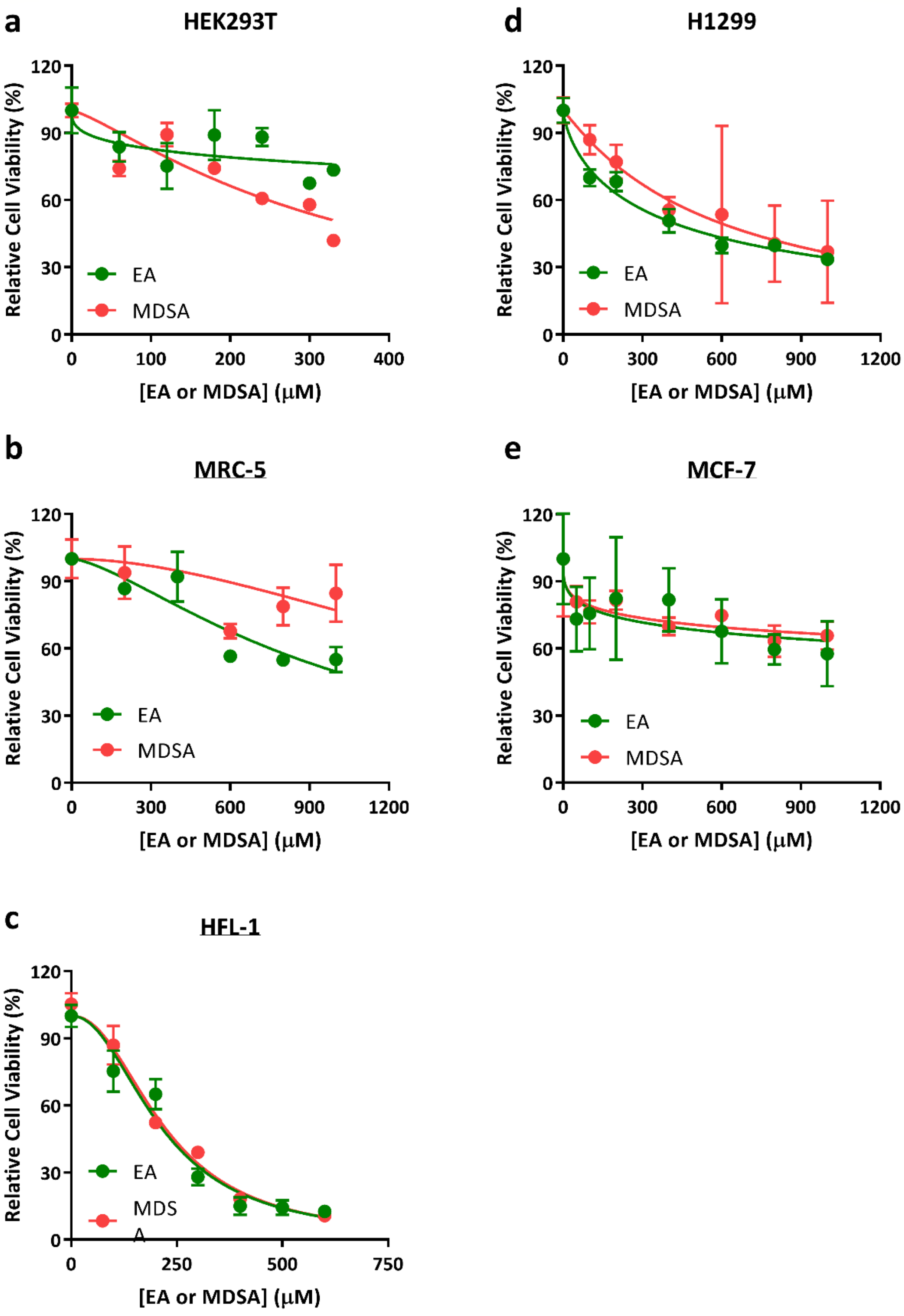

Figure S12. Cell viability after treatment with EA or MDSA. Cell viability was determined using celllltitler-fluor^{TM} assays, at various concentrations of EA or MDSA. N = 3 in each group, mean ± SD. **a** HEK293T, **b** MRC-5, **c** HFL-1, **d** H1299, and **e** MCF-7 cells.

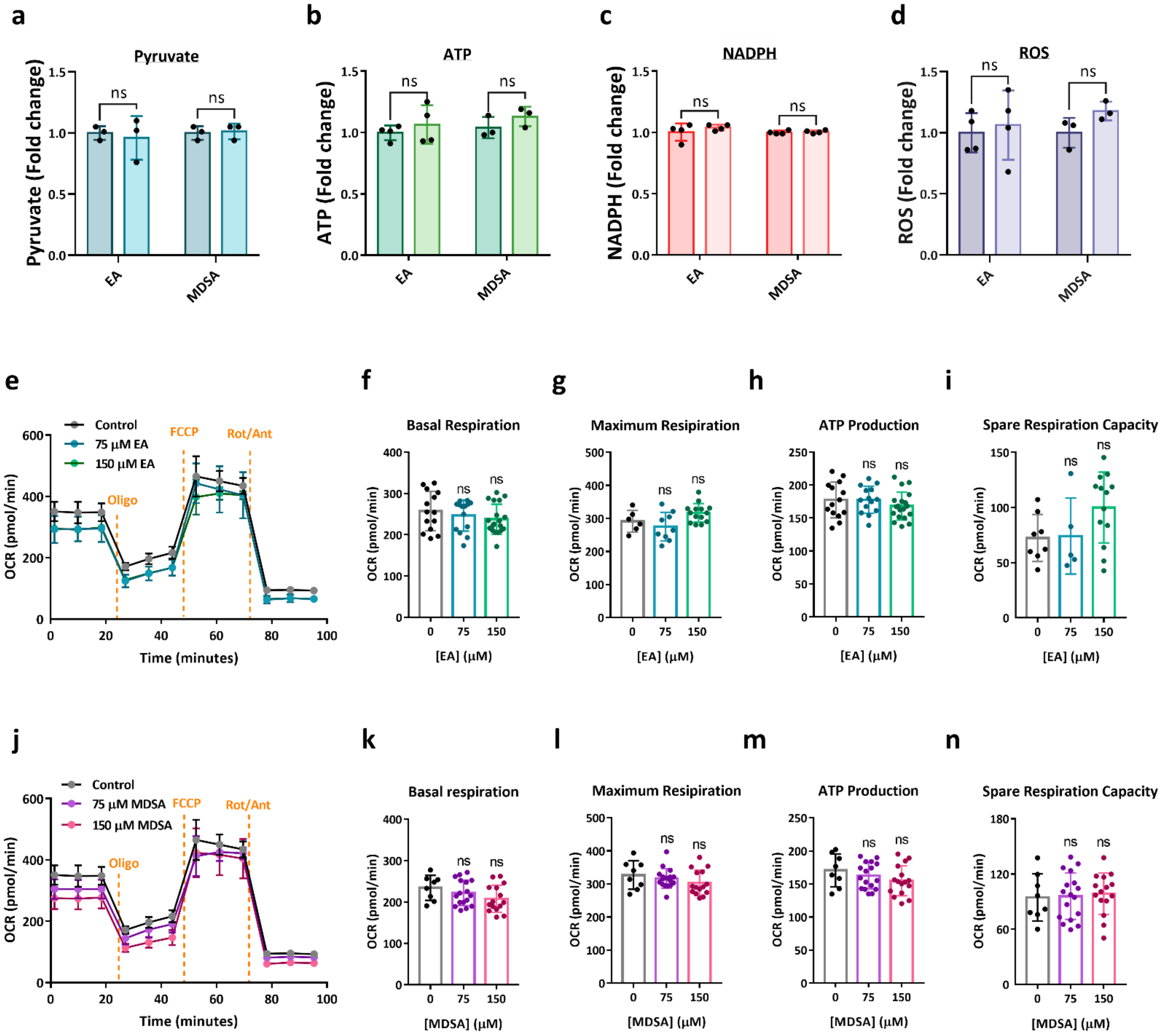

Figure S13. Changes in the levels of pyruvate, ATP, NADPH, ROS, and oxygen consumption rate (OCR) in MCF-7 cells treated with EA or MDSA. The proportional change of cellular pyruvate, ATP, NADPH, and ROS in MCF-7 cells in the presence of EA or MDSA (0, 75, and 150 μM). **a** The fold change in pyruvate levels. N = 3. Unpaired Student's *t*-test. ns, no statistical significance. **b** The fold change in ATP levels. N = 3-4. Unpaired Student's *t*-test. ns, no statistical significance. **c** The fold change in NADPH levels. N = 4. Unpaired Student's *t*-test. ns, no statistical significance. **d** The fold change in ROS levels. N = 3-4. Unpaired Student's *t*-test. ns, no statistical significance. **e** The oxygen consumption rate in EA-treated MCF-7 cells. N = 3. **f** The basal respiration rate. N = 14, 14 and 19, from left to right. One-way ANOVA with Dunnett's test. ns, no statistical significance. **g** The maximal respiration rate. N = 14, 14 and 19, from left to right. One-way ANOVA with Dunnett's test. ns, no statistical significance. **h** ATP production. N = 14, 14 and 19, from left to right. One-way ANOVA with Dunnett's test. ns, no statistical significance. **i** The spare respiration capacity. N = 8, 5 and 13, from left to right. One-way ANOVA with Dunnett's test. ns, no statistical significance. **j** The oxygen consumption rate in MDSA-treated MCF-7 cells (N = 3-4). **k** The basal respiration rate. N = 8, 17 and 15, from left to right. One-way ANOVA with Dunnett's test. ns, no statistical significance. **l** The maximal respiration rate. N = 8, 17 and 15, from left to right. One-way ANOVA with

Dunnett's test. ns, no statistical significance. **m** ATP production. N = 8, 17 and 15, from left to right. One-way ANOVA with Dunnett's test. ns, no statistical significance. **n** The spare respiration capacity. N = 8, 17 and 15, from left to right. One-way ANOVA with Dunnett's test. ns, no statistical significance. Error bars are mean ± SD. Oligo: Oligomycin; FCCP: Carbonyl cyanide-4 (trifluoromethoxy) phenylhydrazone; Rot/Ant: Rotenone/Antimycin A.

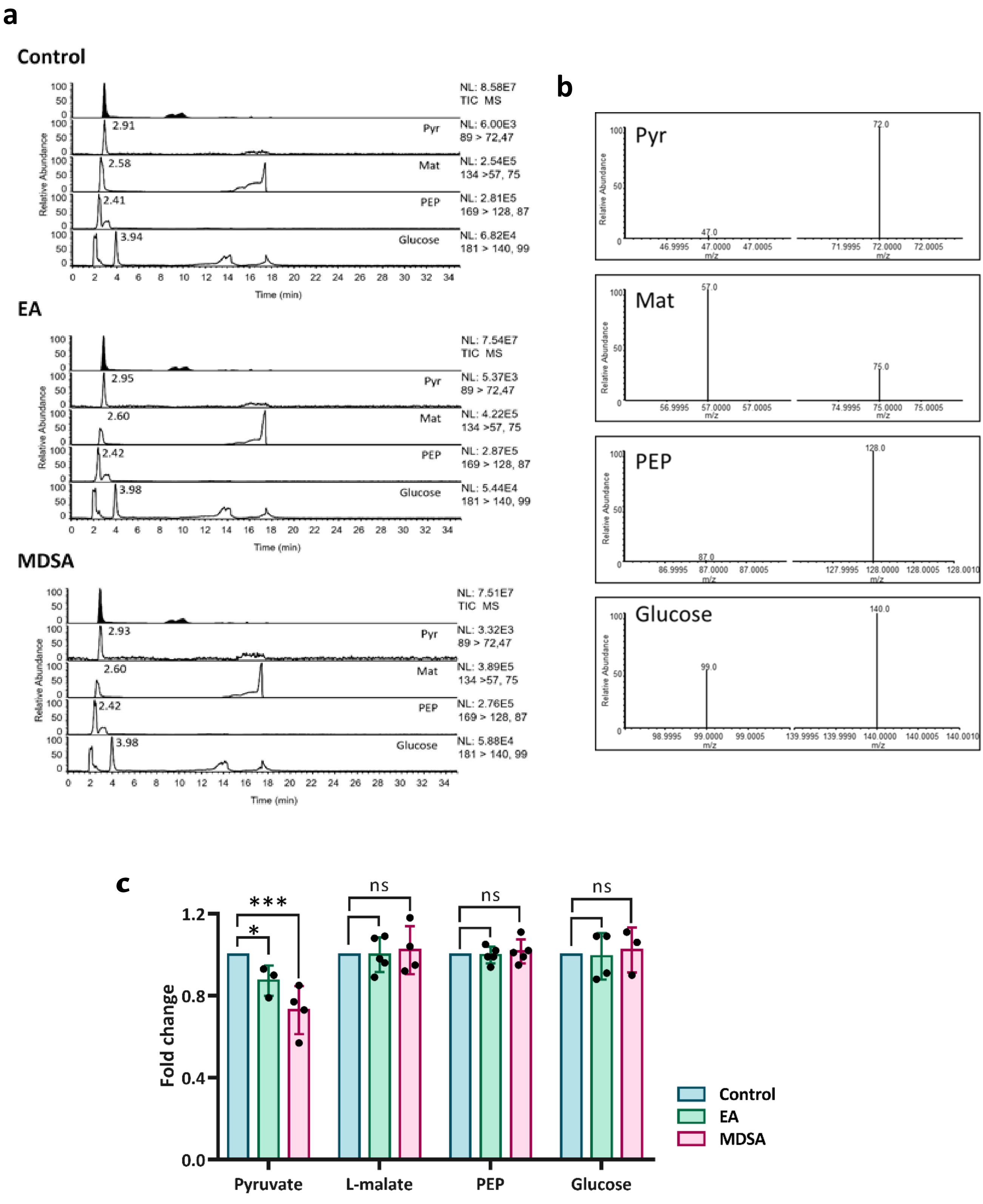

Figure S14. Fold change in the cellular levels of pyruvate, L-malate, PEP and glucose in the presence of allosteric inhibitors EA or MDSA. Metabolite analysis was carried out by mass spectrometry. **a** LC-ESI-MS/MS with SRM analysis of cellular pyruvate, L-malate, PEP and glucose. **b** Mass spectra of pyruvate, L-malate, PEP and glucose. **c** The bar graphs depict the fold change in pyruvate, L-malate, PEP and glucose in EA-and MDSA-treated HEK293T cells. N = 3-5**.** Unpaired Student's *t*-test. **p* < 0.05, and ****p*< 0.001. ns, no statistical significance. Error bars are mean ± SD.

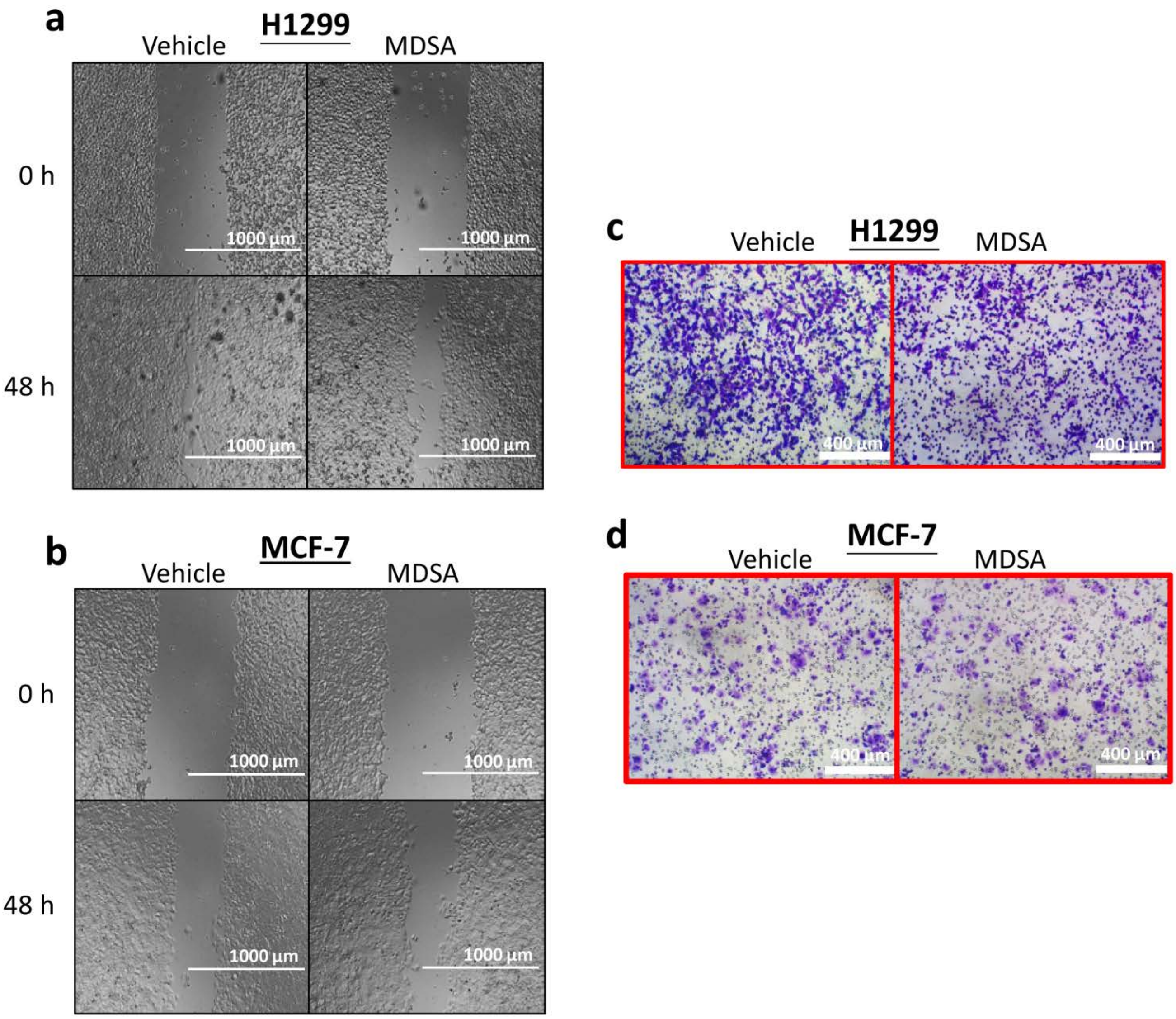

Figure S15. The effect of MDSA on the migration and invasion of H1299 and MCF-7 cells. a and **b** Migration (Wound healing assay) of H1299 and MCF-7 cells in the presence of MDSA, respectively. **c** and **d** Invasion assay of H1299 and MCF-7 cells in the presence of MDSA, respectively.

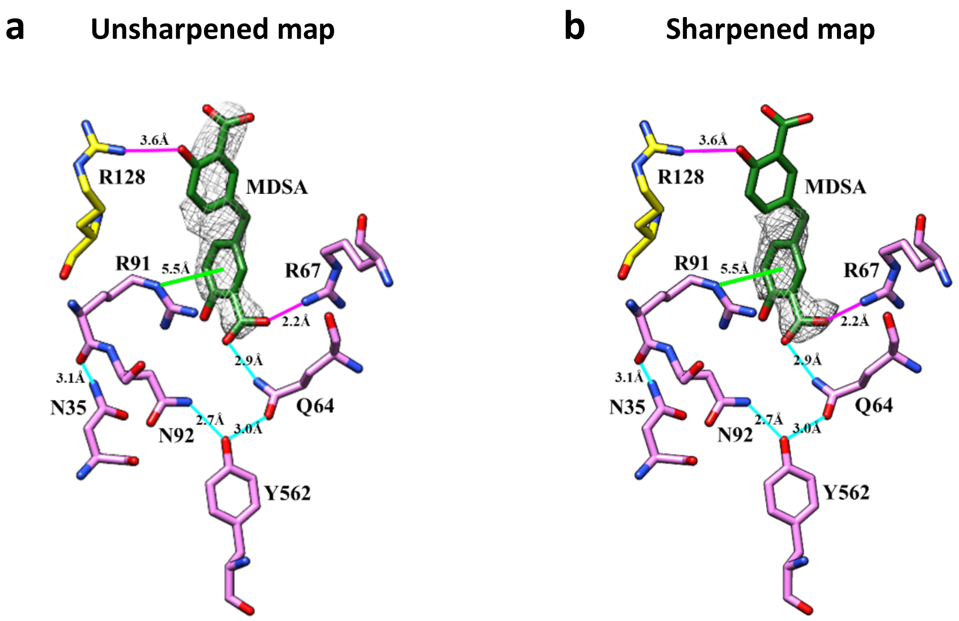

Figure S16. Allosteric site coordination of ME2-MDSA. The structures illustrate the interactions between MDSA and ME2 surrounding the allosteric site from **a** unsharpened map contoured at 7.5 σ and **b** sharpened map contoured at 8.5 σ, respectively.

Table S1. Disalicylic acid and naphthoic acid derivatives as inhibitors of human ME2

Compound	IC_{50} (μM)	Chemical Structure
5,5'-Methylenedisalicylic acid (MDSA)	0.51 ± 0.03	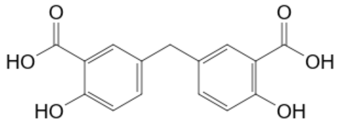
4,4'-Methylene-bis(3-hydroxy-2-naphthoic acid)<br>Embonic Acid (EA)	1.14 ± 0.05	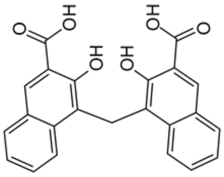
Salicylic acid	800.7 ± 125.6	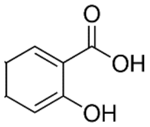
3-Benzoylbenzoic acid	652.1 ± 45.0	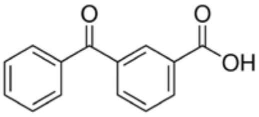
3,5-Dihydroxy-2-naphthoic acid	12.4 ± 1.1	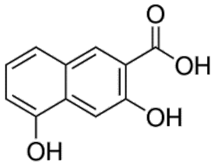
3,7-Dihydroxy-2-naphthoic acid	37.6 ± 4.3	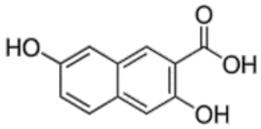
7-Bromo-3-hydroxy-2-naphthoic acid	307.8 ± 19.4	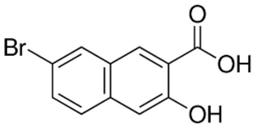
1-Hydroxy-2-naphthoic acid	74.7 ± 6.8	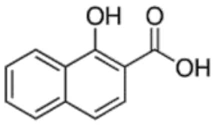
3-Hydroxy-2-naphthoic acid	105.4 ± 4.7	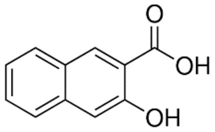
2,6-Dicarboxynaphthalene	352.9 ± 26.2	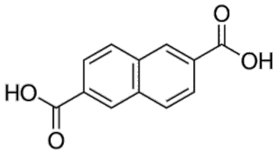

Table S2. Cryo-EM data collection, refinement and validation statistics

	ME2-EA (EMD-33146) (PDB-7XDF)	ME2-MDSA (EMD-33147) (PDB-7XDG)	ME2-open form (EMD-33145) (PDB-7XDE)
Data collection			
EM equipment	Titan Krios	Titan Krios	Titan Krios
Voltage (kV)	300	300	300
Cs (mm)	2.7	2.7	2.7
Magnification (nominal)	165,000	165,000	81,000
Detector	K2	K2	K3
Pixel size (Å)	0.82	0.82	1.061
Electron exposure ($e^-/Å^2$)	~ 50	~ 50	~ 40
Exposure time (s)	4.5	4.5	2.8
Frames (no.)	60	60	40
Defocus range (μm)	-0.5 ~ -2.9	-0.5 ~ -3.3	-0.5 ~ -3.5
Reconstruction			
Software	Relion & cryoSPARC	Relion & cryoSPARC	Relion & cryoSPARC
Micrographs stacks (no.)	20,077	8,842	5,091
Final particle images (no.)	75,943	44,386	90,061
Symmetry imposed	I	I	I
Map final resolution (Å)*	2.72	2.84	2.72
Map sharpening B-factor ($Å^2$)	-86.6	-80.8	-117.1
Atomic modeling			
Software	Coot & Phenix	Coot & Phenix	Coot & Phenix
Number of protein residues†	2,204	2,204	2,248
Number of ligands†	EA: 4<br>NAD^+: 8	MDSA: 4<br>NAD^+: 8	NAD^+: 4
Number of atoms†	17,868	17,836	17,896
Map CC (around atoms) ‡	0.84	0.82	0.81
RMSD bond lengths (Å)	0.005	0.005	0.007
RMSD bond angles (°)	0.981	0.963	1.073
Clash score‡	12.06	12.12	8.42
Ramachandran favored (%)‡	98.54	98.50	98.75
Ramachandran allowed (%)‡	1.46	1.50	1.25
Ramachandran outliers (%)‡	0	0	0
Rotamer outliers (%)‡	0	0	0
C_β deviations‡	0	0	0
MolProbity score‡	1.59	1.60	1.46
EMRinger score	4.34	3.75	3.27

*According to FSC=0.143; †Statistics are given for one icosahedral asymmetric unit

‡According to the criterion of Chen *et al.*, 2010 [2]

Table S3. Kinetic parameters of the inhibitor-binding site mutants of human ME2

ME2		$K_{m,NAD}$ (mM)	$K_{0.5,Malate}$ (mM)	h	k_{cat} (s^{-1})
WT	(-)	0.83 ± 0.08	15.34 ± 2.44	1.77 ± 0.33	202.7 ± 5.8
	(+)	0.31 ± 0.01	3.10 ± 0.46	1.16 ± 0.19	267.4 ± 2.0
N35A	(-)	2.23 ± 0.25	25.94 ± 10.83	1.00 ± 0.13	69.5 ± 3.5
	(+)	1.71 ± 0.13	22.91 ± 4.32	1.20 ± 0.10	149.5 ± 4.7
N35D	(-)	0.22 ± 0.01	2.68 ± 0.79	1.00 ± 0.29	9.7 ± 0.1
	(+)	0.26 ± 0.33	2.32 ± 0.83	1.00 ± 0.39	9.7 ± 0.2
N35Q	(-)	0.60 ± 0.06	39.94 ± 12.12	1.20 ± 0.08	26.5 ± 0.8
	(+)	0.62 ± 0.08	11.28 ± 2.83	1.24 ± 0.16	29.5 ± 1.1
N57A	(-)	5.04 ± 0.88	93.40 ± 32.59	1.57 ± 0.23	57.8 ± 3.2
	(+)	1.52 ± 0.18	40.11 ± 12.65	1.00 ± 0.14	141.4 ± 4.5
N57S	(-)	0.50 ± 0.03	18.68 ± 5.22	1.07 ± 0.16	93.5 ± 1.4
	(+)	0.30 ± 0.02	4.07 ± 0.41	1.12 ± 0.12	123.3 ± 1.8
E59A	(-)	0.86 ± 0.36	25.16 ± 4.89	1.11 ± 0.09	25.6 ± 3.4
	(+)	1.29 ± 0.27	17.22 ± 7.48	1.22 ± 0.30	34.5 ± 2.7
E59N	(-)	0.41 ± 0.09	18.92 ± 4.57	1.41 ± 0.26	59.2 ± 3.1
	(+)	0.47 ± 0.06	13.09 ± 2.05	1.00 ± 0.08	61.0 ± 1.8
Q64A	(-)	2.39 ± 0.47	21.36 ± 4.11	1.03 ± 0.21	31.4 ± 2.8
	(+)	1.29 ± 0.23	11.73 ± 0.98	1.00 ± 0.05	90.0 ± 5.9
Q64E	(-)	0.91 ± 0.14	13.29 ± 4.45	1.00 ± 0.20	6.6 ± 0.9
	(+)	0.84 ± 0.13	14.01 ± 0.15	1.00 ± 0.18	6.5 ± 1.0
Q64N	(-)	0.35 ± 0.04	18.90 ± 5.97	1.09 ± 0.14	12.8 ± 2.0
	(+)	0.39 ± 0.05	16.07 ± 9.26	1.00 ± 0.24	10.8 ± 2.7
R67A	(-)	0.32 ± 0.05	4.23 ± 1.40	1.00 ± 0.31	6.2 ± 0.2
	(+)	0.24 ± 0.07	4.92 ± 0.97	1.00 ± 0.17	5.3 ± 0.3
R91A	(-)	1.87 ± 0.18	10.66 ± 2.28	1.34 ± 0.21	33.3 ± 3.5
	(+)	1.68 ± 0.28	10.52 ± 2.31	1.33 ± 0.21	32.1 ± 3.4
N92A	(-)	3.48 ± 0.58	27.99 ± 14.44	1.00 ± 0.19	61.2 ± 5.3
	(+)	1.58 ± 0.10	3.49 ± 0.92	1.00 ± 0.25	152.6 ± 3.7
N92Q	(-)	1.15 ± 0.15	29.01 ± 13.77	1.19 ± 0.16	133.2 ± 4.6
	(+)	1.19 ± 0.09	17.12 ± 3.89	1.15 ± 0.10	172.1 ± 6.3
R128A	(-)	1.95 ± 0.31	3.91 ± 0.57	2.03 ± 0.59	17.3 ± 0.9
	(+)	2.17 ± 0.37	4.42 ± 0.59	1.72 ± 0.37	19.3 ± 1.2
Y562A	(-)	0.44 ± 0.07	22.85 ± 7.42	1.00 ± 0.12	8.0 ± 0.3
	(+)	0.41 ± 0.07	14.83 ± 6.58	1.00 ± 0.21	7.4 ± 0.3

*(-), no fumarate added; (+), with 5 mM fumarate.

Table S4. Mutagenic primers used in this study

ME2 mutant	5' to 3'
N35A_F	GCTGAACCCAAGAACAGCGAAGGGAATGGCATTTAC
N35A_R	GTAAATGCCATTCCCTTCGCTGTTCTTGGGTTCAGC
N35D_F	GCTGAACCCAAGAACAGACAAGGGAATGGCATTTAC
N35D_R	GTAAATGCCATTCCCTTGTCTGTTCTTGGGTTCAGC
N35Q_F	GCTGAACCCAAGAACACAGAAGGGAATGGCATTTAC
N35Q_R	GTAAATGCCATTCCCTTCTGTGTTCTTGGGTTCAGC
K57A_F	CTTCAAGGACTTCTACCTCCCGCGATAGAGACACAAGATATTC
K57A_R	GAATATCTTGTGTCTCTATCGCGGGAGGTAGAAGTCCTTGAAG
K57S_F	CTTCAAGGACTTCTACCTCCCTCTATAGAGACACAAGATATTC
K57S_R	GAATATCTTGTGTCTCTATAGAGGGAGGTAGAAGTCCTTGAAG
E59A_F	CTACCTCCCAAAATAGCGACACAAGATATTCAAGCC
E59A_R	GGCTTGAATATCTTGTGTCGCTATTTTGGGAGGTAG
E59N_F	GGACTTCTACCTCCCAAAATAAACACACAAGATATTCAAGCC
E59N_R	GGCTTGAATATCTTGTGTGTTTATTTTGGGAGGTAGAAGTCC
Q64A_F	CAAAATAGAGACACAAGATATTGCGGCCTTACGATTTCATAGAAAC
Q64A_R	GTTTCTATGAAATCGTAAGGCCGCAATATCTTGTGTCTCTATTTTG
Q64E_F	CAAAATAGAGACACAAGATATTGAAGCCTTACGATTTCATAGA
Q64E_R	TCTATGAAATCGTAAGGCTTCAATATCTTGTGTCTCTATTTTG
Q64N_F	CAAAATAGAGACACAAGATATTAACGCCTTACGATTTCATAGAAAC
Q64N_R	GTTTCTATGAAATCGTAAGGCGTTAATATCTTGTGTCTCTATTTTG
R67A_F	CACAAGATATTCAAGCCTTAGCGTTTCATAGAAACTTGAAG
R67A_R	CTTCAAGTTTCTATGAAACGCTAAGGCTTGAATATCTTGTG
R91A_F	CTACATAATGGGAATACAAGAAGCGAATGAGAAATTGTTTTATAG
R91A_R	CTATAAAACAATTTCTCATTCGCTTCTTGTATTCCCATTATGTAG
N92A_F	CATAATGGGAATACAAGAAAGAGCGGAGAAATTGTTTTATAGAATAC
N92A_R	GTATTCTATAAAACAATTTCTCCGCTCTTTCTTGTATTCCCATTATG
N92Q_F	CATAATGGGAATACAAGAAAGACAGGAGAAATTGTTTTATAGAATAC
N92Q_R	GTATTCTATAAAACAATTTCTCCTGTCTTTCTTGTATTCCCATTATG
R128A_F	CAGTATGGACACATCTTTGCGAGACCTAAGGGATT
R128A_R	AATCCCTTAGGTCTCGCAAAGATGTGTCCATACTG
Y562A_F	GAACATGGCGGAGTGAAGCGGATTCCCTGCTGCCAG
Y562A_R	CTGGCAGCAGGGAATCCGCTTCACTCCGCCATGTTC

SUPPLEMENTARY REFERENCES

1. Pettersen, E. F. *et al.* UCSF Chimera--a visualization system for exploratory research and analysis. *J Comput Chem* **25**, 1605–12 (2004).
2. Chen, V. B. *et al.* MolProbity: all-atom structure validation for macromolecular crystallography. *Acta Crystallogr. D Biol. Crystallogr.* **66**, 12–21 (2010).
