# Supplementary material for: Suppression of the human malic enzyme 2 modifies energy metabolism and inhibits cellular respiration
Source: Commun Biol. 2023 May 22;6:548. doi: 10.1038/s42003-023-04930-y (PMC10202922; doi:10.1038/s42003-023-04930-y)
Supplement: Supplementary file 2 — Description of Additional Supplementary Files [file 42003_2023_4930_MOESM2_ESM.pdf]

Description of Additional Supplementary Files

File name: Supplementary Data

Description: The source data behind the graphs and charts in the paper.
